# Gut dysbiosis induces the development of depression-like behavior through abnormal synapse pruning in microglia-mediated by complement C3

**DOI:** 10.1186/s40168-024-01756-6

**Published:** 2024-02-20

**Authors:** Wenzhi Hao, Qingyu Ma, Lu Wang, Naijun Yuan, Hua Gan, Liangliang He, Xiaojuan Li, Junqing Huang, Jiaxu Chen

**Affiliations:** 1 Guangzhou Key Laboratory of Formula-Pattern of Traditional Chinese Medicine, Jinan University, Guangzhou, China; 2 School of Traditional Chinese Medicine, Beijing University of Chinese Medicine, Beijing, China

**Keywords:** Complement C3, Depression, Fecal microbiota transplantation, Gut microbiota, Microglia, Synaptic pruning

## Abstract

**Background:**

Remodeling eubiosis of the gut microenvironment may contribute to preventing the occurrence and development of depression. Mounting experimental evidence has shown that complement C3 signaling is associated with the pathogenesis of depression, and disruption of the gut microbiota may be an underlying cause of complement system activation. However, the mechanism by which complement C3 participates in gut-brain crosstalk in the pathogenesis of depression remains unknown.

**Results:**

In the present study, we found that chronic unpredictable mild stress (CUMS)-induced mice exhibited obvious depression-like behavior as well as cognitive impairment, which was associated with significant gut dysbiosis, especially enrichment of Proteobacteria and elevation of microbiota-derived lipopolysaccharides (LPS). In addition, peripheral and central complement C3 activation and central C3/CR3-mediated aberrant synaptic pruning in microglia have also been observed. Transplantation of gut microbiota from CUMS-induced depression model mice into specific pathogen-free and germ-free mice induced depression-like behavior and concomitant cognitive impairment in the recipient mice, accompanied by increased activation of the complement C3/CR3 pathway in the prefrontal cortex and abnormalities in microglia-mediated synaptic pruning. Conversely, antidepressants and fecal microbiota transplantation from antidepressant-treated donors improved depression-like behaviors and restored gut microbiome disturbances in depressed mice. Concurrently, inhibition of the complement C3/CR3 pathway, amelioration of abnormal microglia-mediated synaptic pruning, and increased expression of the synapsin and postsynaptic density protein 95 were observed. Collectively, our results revealed that gut dysbiosis induces the development of depression-like behaviors through abnormal synapse pruning in microglia-mediated by complement C3, and the inhibition of abnormal synaptic pruning is the key to targeting microbes to treat depression.

**Conclusions:**

Our findings provide novel insights into the involvement of complement C3/CR3 signaling and aberrant synaptic pruning of chemotactic microglia in gut-brain crosstalk in the pathogenesis of depression.

Video Abstract

**Supplementary Information:**

The online version contains supplementary material available at 10.1186/s40168-024-01756-6.

## Background

According to the World Health Organization (WHO), nearly a billion people, including 14% of the world’s adolescents, were living with mental disorders as of 2019. More than 240 million people are affected by depression, and this has increased by more than 25% in the first year of the COVID-19 pandemic [1]. Gut-brain crosstalk links emotional disorders and cognitive centers of the brain with peripheral control and function of the gastrointestinal tract. Cross-communication between the gut and brain occurs via complex functions of neuronal, hormonal, and immune reflexes. Under the effects of medicines and phytochemicals, intestinal microbes may drive sensory signals from the gut to the central nervous system (CNS) and influence neurons, synapses, and neuroimmunity to inhibit the occurrence and progression of encephalopathy [2, 3]. Recent research suggests that depression is associated with dysregulation of the gut microbiota [4]. Gut microbiota-derived metabolites are key regulators of the microbial-mediated development of depression [5, 6]. In addition, manipulation of the gut microbiota has the potential for depression therapy, such as fecal microbiota transplantation (FMT) [7].

Synaptic pruning is an important link in the formation of correct neural circuits and is crucial for brain development and maintenance of neural homeostasis in depression [8, 9]. Recent research has revealed that complement is involved in developmental synaptic pruning and pathological synapse loss, the latter of which contributes to various brain diseases including schizophrenia, depression, autism, and anxiety disorders [10, 11]. In the brain, the complement system participates in the regulation of synaptic pruning in microglia [12]. Complement C3 is the central link in the complement activation pathway [13]. Through complement labeling at the synapse, complement C3 binds to complement receptors, mainly complement receptor 3 (CR3), on chemotactic microglia, inducing microglia to engulf the labeled cellular components [14]. Both peripherally and centrally activated complement systems have been observed in patients with depression and animal models of depression [15]. These results indicate that complement C3/CR3 signaling is associated with the pathogenesis of depression. However, the source of complement C3 has not been determined [15]. The gut microbiota modulates host peripheral and neuroimmunity, and its microbial metabolites are important activators of complement C3 [16]. However, whether the gut microbiota is the source of the complement that mediates central synaptic pruning during the pathogenesis of depression and whether the complement is an important link in gut-brain crosstalk is still unknown.

Numerous epidemiological studies have shown that the intake of phytochemicals from herbal medicines in the form of pharmaceuticals or nutraceuticals has an impact on the risk of depression [17, 18]. Xiaoyaosan (XYS) is a medicine comprising *Bupleurum chinense* DC*.*, *Paeonia lactiflora* Pall*.*, *Angelica sinensis* (Oliv.) Diels, *Atractylodes lancea* (Thunb.) DC*.*, *Wolfiporia extensa* (Peck) Ginns.* (syn. Poria cocos* (Schwein.) F.A. Wolf), *Glycyrrhiza glabra* L., *Mentha canadensis* L., and *Zingiber officinale* Roscoe in a 5:5:5:5:5:4:1:5 ratio [19, 20]. XYS has a long history as a traditional treatment for emotional disorders in Asian countries, including China, Japan, and South Korea, and has also been used for traditional application as a health food or herbal medicine in European counties, including the UK, the Netherlands, and Germany for > 30 years [21, 22]. Furthermore, all herbs used in XYS are listed in the European Pharmacopoeia (partially listed in the British Pharmacopoeia), edible, and used as dietary supplements or nutritional health products worldwide [22, 23]. It has been reported that XYS alleviates depression accompanied by regulation of gut microecology [24]. Current research suggests that gut microbes contribute to the metabolism of XYS polyphenols to exert anti-neuroinflammatory effects [18]. However, the dominant pathway of gut-brain crosstalk in XYS-mediated alleviation of depression is still unclear.

In this study, we used XYS, a potential antidepressant drug, to explore the role of gut microbiota in the treatment of depression, as well as the specific mechanism of the interaction between gut microbes and the CNS. We hypothesize that depression is caused by dysbiosis of the gut microbiota and abnormal synaptic pruning of microglia mediated by complement C3. In addition, the attenuation of depression by XYS is primarily mediated through modulation of the gut microbiota and microbial metabolites, which subsequently leads to amelioration of the complement system and abnormal synaptic pruning. Therefore, the changes in the gut microbiota, complement system, and synaptic pruning during the development of depression and palliative effects of XYS administration in a murine model of chronic unpredicted mild stress (CUMS)-induced depression were evaluated. Antibiotic intervention was used to explore the effect of microbiota clearance on antidepressant efficacy. Next, we interfered with the intestinal microbial flora of normal and depressed specific pathogen-free (SPF) mice using CUMS-FMT and XYS-FMT, respectively, to explore the therapeutic effect and regulatory mechanism of intestinal microbial flora on depressive behavior. Finally, we performed FMT in germ-free (GF) mice to further verify the crosstalk between gut microbes and synaptic pruning, further demonstrating that the gut microbiota is integral to depression remission.

## Methods

### Animals and CUMS procedure

Six- to eight-week-old SPF male C57BL/6 mice (SYXK (Yue) 2017–0174) weighing 20 ± 2 g were subjected to a 7-day adaptive feeding process. The experimental conditions included a room temperature of 21 ± 2 °C, relative humidity of 30–40%, and 12 h/12 h light/dark cycle. Eight-week-old GF male C57BL/6 mice (SYXK (Yue) 2020–0233) were strictly fed in a sterile environment (Germ-free Laboratory Animal Center of the First Affiliated Hospital of Sun Yat-sen University). All mice in the study were individually housed.

The CUMS depression model was induced by daily exposure to alternating stressors for a continuous period of 8 weeks, as previously described [25]. The stressors included 24 h of food and water deprivation, 5 min of heat stress by placing the mice in an oven at 45 °C, 5 min of ice-water swimming at 4–8 °C, 3 h under body restraint, 2 min of tail pinching, day and night reversal, and other stimuli. The objective of employing CUMS modeling in our study is to replicate the chronic low-intensity stress experienced by individuals in their daily lives. This modeling approach aims to introduce continuous and unpredictable mild stimuli, with the key emphasis on ensuring that the sequence of stimuli remains random and non-repetitive, thereby preventing animals from predicting the occurrence of stressors. To prevent mice from predicting the occurrence of the stimuli, the stressors were randomly distributed, and the same stressors were separated by at least 7 days (Additional file 2: Supplementary Table 1) [25].

### Treatments and sample collection

**Fig. 1:**
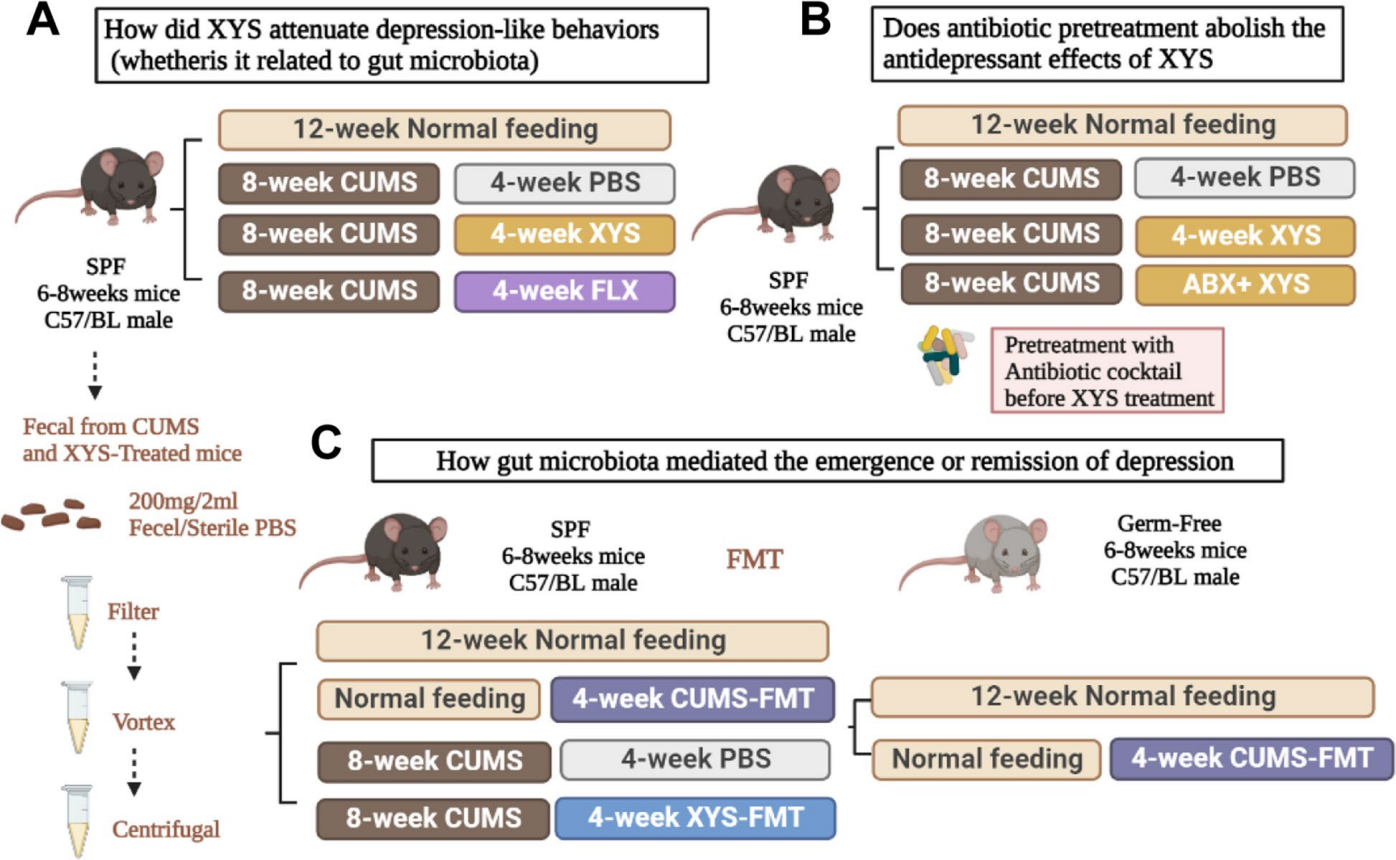
Study design for the whole experiment. *SPF* Specific pathogen-free, *PBS *Phosphate-buffered saline, *XYS* Xiaoyaosan, *CUMS* Chronic unpredicted mild stress, *FLX* Fluoxetine, *ABX* Antibiotics, *FMT* Fecal microbiota transplantation

The experimental design is illustrated in Fig. 1. The changes in the gut microbiota, complement system, and synaptic pruning during the development of depression, and the pharmacodynamic role of XYS as a potential antidepressant were assessed (Fig. 1a). After 1 week of acclimation, mice were randomly divided into four groups: control, CUMS, CUMS + XYS, and CUMS + fluoxetine (FLX). Except for the control group, the other three groups were subjected to a variety of stressors for eight consecutive weeks. The treatments were as follows: (1) control group: no stress stimulation was administered and served as a negative control; (2) CUMS group: CUMS for 8 weeks, followed by daily oral administration of phosphate-buffered saline (PBS) for 4 weeks; (3) CUMS + XYS group: CUMS for 8 weeks, followed by daily oral administration of XYS (0.658 g/kg/day; batch number: 20190724; Jiuzhitang Group Co. Ltd) for 4 weeks; and (4) CUMS + FLX group: CUMS for 8 weeks, followed by daily oral administration of FLX (20 mg/kg/day; F844356; Macklin) for 4 weeks. Body weight was measured weekly during the entire duration of the study. The mice were immediately sacrificed after the last behavioral test, and fecal samples of all mice were collected sterilely and stored at − 80 °C for future analysis. Serum was obtained by centrifuging blood samples at 3000 rpm for 15 min at 4 C and stored at − 80 °C. Colons were flushed with PBS and fixed in 10% formalin for subsequent histological analysis; the remaining colon was fixed in electron microscopy fluid for subsequent experiments. Hippocampus, hypothalamus, and prefrontal cortex tissues were collected for future analysis. Fresh feces (gut microbiota with or without metabolites) in the CUMS and CUMS + XYS groups were collected for subsequent FMT (Fig. 1A).

The ability of antibiotics to interfere with the antidepressant effects of XYS was assessed (Fig. 1B). The effect of antibiotic-induced microbiota disruption on the efficacy of XYS was assessed by pre-disrupting gut microbes with an antibiotic cocktail consisting of ampicillin (1.5 g/mL; A9518; Sigma Aldrich), vancomycin (500 mg/L; V820413; Macklin), ciprofloxacin (200 mg/L; C9371; Solarbio), imipenem (250 mg/L; S27995), and metronidazole (1 g/L; B1976; Ape) in the mice’s daily drinking water (1 week; to prevent stress induced by oral gavage in mice) prior to the 4-week XYS administration.

The therapeutic effects of fecal microbiota in depression were evaluated using 6- to 8-week-old SPF and GF male C57BL/6 mice (Fig. 1C). After 1 week of acclimation, SPF mice were randomly divided into four groups as follows: (1) control group: no stress stimulation was received, and served as a negative control; (2) Control + FMT(CUMS) group: no stress stimulation for 8 weeks, followed by daily FMT (feces from CUMS group in experiment 1) for 4 weeks; (3) CUMS group: CUMS for 8 weeks, followed by daily oral administration of PBS for 4 weeks; and (4) CUMS + FMT(XYS) group: CUMS for 8 weeks, followed by daily FMT (feces from CUMS + XYS group in experiment 1) for 4 weeks. After 1 week of acclimation, GF mice were randomly divided into two groups as follows: (1) GF group: no stress stimulation was received, and served as a negative control; and (2) GF + FMT(CUMS) group: no stress stimulation for 8 weeks, followed by daily FMT (feces from CUMS group in experiment 1) for 4 weeks.

Body weight was measured weekly during the entire study. The mice were immediately sacrificed after the final behavioral test, and fecal samples from all mice were collected and stored at − 80 °C for future analysis. Serum was obtained by centrifuging blood samples at 3000 rpm for 15 min at 4 C and stored at − 80 C. Colons were flushed with PBS and fixed in 10% formalin for subsequent histological analysis; the remaining colon was fixed in electron microscopy fluid for subsequent experiments. Hippocampus, hypothalamus, and prefrontal cortex tissues were collected for future analysis.

### Behavioral tests

#### Sucrose preference test (SPT)

The susceptibility of mice to rewards was measured using a sucrose preference test (SPT) to assess the degree of depression. The SPT includes two periods, training and testing [26]. In this study, the SPT included a 72-h training period. Initially, mice were provided with two bottles of 1% sucrose solution for 24 h. Subsequently, mice were presented with one bottle of 1% sucrose solution and one bottle of pure water for the next 24 h. Finally, the positions of the sucrose and water bottles were switched for an additional 24 h. After a 72-h training experiment and 24 h of food and water deprivation, the mice were given access to a bottle of pure water and a bottle of 1% sucrose solution simultaneously. One hour later, the volumes of the remaining pure water and sucrose solutions were recorded. The SPT was conducted before subjecting mice to CUMS (as a baseline measurement) and after the CUMS experiment was completed.

#### Open field test (OFT)

The open field behavioral test method is used to observe autonomous behavior, exploratory behavior, and stress in experimental animals in an unaccustomed environment. The OFT was performed at weeks 0, 8, and 12 in our study. The mice were placed in a behavioral operating room for 10 min for adaptation and then moved to the center zone. The camera recording was initiated and timed, and the behavior of the mice was observed for 5 min. Immediately after each experiment, the boxes were cleaned with 75% alcohol. According to previous study, OFT was conducted under dim light conditions, specifically at 60 lx [27]. The OFT was performed using the internationally recognized Behavior Analysis software (EthoVision software analysis system, Noldus Information Technology, Leesburg, VA, USA) and included the analysis of the total movement distance of each group of mice.

#### Tail suspension test (TST)

Mice were suspended on a horizontal rod 50 cm from the ground, with their tails fixed using adhesive tape. The experimental period lasted for 6 min, of which the activity of each mouse was recorded in the final 4 min. Immobility time (s) was defined as the time required for mice to give up struggling and remain completely motionless. Immediately after each experiment, the boxes were cleaned with 75% alcohol. The TST experimental data were analyzed using Behavior Analysis software (EthoVision software analysis system, Noldus Information Technology, Leesburg, VA, USA).

#### Elevated plus-maze test (EPM)

The EPM experiment was performed using a plus-maze device consisting of two open arms, two closed arms, and a central platform. The dimensions of the open and closed arms were both 30 × 7 cm; the closed arms were covered by 20-cm baffles on both sides. In accordance with previous studies, the EPM experiments were conducted under dim light conditions, precisely at an intensity of 60 lx [27]. Mice were placed individually in the central area (7 × 7 cm), 60 cm above the ground, and their movements were recorded for 5 min. Following each experiment, the enclosures were promptly cleaned using a 75% alcohol solution. The results of the EPM test were analyzed using Behavior Analysis software (EthoVision software analysis system, Noldus Information Technology, Leesburg, VA, USA).

#### Novel object recognition test (NORT)

The novel object recognition test is used to determine learning and memory abilities in rodents. The experiment was divided into three phases: training, recognition training, and testing. In stage 1, the mouse was taken out of the cage, placed in the middle of the open field box with its back facing the operator, and allowed to explore freely for 10 min. In stage 2, two identical objects (old objects) were placed in the relative object limit of the open field box, the mouse was removed from the cage 24 h after phase 1, placed on the same object as the two objects, and allowed to explore freely for 10 min in the center of the open field box from a distance. In stage 3, an object (old object) and a novel object were placed in the open field box at the object limit for recognition training. After recognition training for 24 h, the mouse was placed between the new and old object and allowed to explore freely for 10 min. Subsequent to each experimental session, the enclosures were promptly cleaned using a 75% alcohol solution. NORT data were analyzed using Behavior Analysis software (EthoVision software analysis system, Noldus Information Technology, Leesburg, VA, USA). The time the mouse spent exploring each object (time spent exploring the familiar object, N1; time spent exploring the novel object, N2) was recorded. The discrimination ratio (DR, %) was calculated as follows: DR = N2/(N2 + N1) × 100.

#### Y-maze task

The Y-maze task is used to determine spatial recognition and memory ability and was used to test short-term memory in this experiment. The Y-maze is a three-arm horizontal maze (30 cm long, 8 cm wide, and 15 cm high) in which the three arms are symmetrically separated at 120°. Mice were placed in a behavioral operating room for 10 min for adaptation and then placed at the end of one arm, blocking one of the arms and allowing free shuttling between the other two arms. The experiment begun 2 h later, and all three arms were opened (the previously blocked arm was defined as the novelty arm). The camera recording was initiated and timed, and the behavior of the mice was observed for 5 min. The boxes were cleaned with 75% alcohol immediately after each experiment. The Y-maze task was performed using Behavior Analysis software (Noldus Information Technology).

### Bioassays

#### Hematoxylin and eosin (HE) staining

Samples of colonic tissue were fixed in 10% formalin, decalcified, dehydrated, made transparent, and then dipped, and embedded in paraffin. Five-micrometer-thick tissue samples were prepared using a microtome. Sections were dewaxed with xylene, passed through an aqueous ethanol series, stained with HE, and observed under a microscope [28].

#### Colon ultrastructural morphology

A segment of the proximal colon was collected and immediately fixed in 2.5% glutaraldehyde solution at 4 °C overnight. Segments were rinsed three times in 1 M phosphate-buffered solution for 15 min each time, dehydrated using graded ethanol, soaked in isoamyl acetate for 20 min twice, and routinely dried and processed. Colonic ultrastructural images were obtained using an electron microscope (SU8100; Hitachi).

#### Enzyme-linked immunosorbent assay (ELISA)

Serum interleukin (IL)-6, IL-10, IL-1β, tumor necrosis factor-α (TNF-α), lipopolysaccharide (LPS), and C3 levels were determined using an ELISA detection kit (Cusabio, Wuhan, China). The IL-6, IL-10, IL-1β, TNF-α, LPS, and C3 levels were determined by measuring the absorbance using a microplate reader and plotting a standard curve.

#### Immunofluorescence (IF) staining

Sections of the prefrontal cortex (PFC) were incubated with primary antibodies overnight and then secondary antibodies in the dark for 1 h. The antibodies used for IF staining were as follows: anti-IBA-1 (1:200, GB12105, Servicebio), anti-CD68 (1:100, GB113109, Servicebio), anti-CR3 (1:500, GB11058, Servicebio), CY3 goat anti-mouse secondary antibody (1:300, GB21303, Servicebio), and 448 AffiniPure Fab Fragment goat anti-rabbit secondary antibody (1:500, GB25303, Servicebio). The brain slices in our study underwent a blocking step using 10% goat normal serum [29]. Samples were counterstained with 4,6-diamidino-2-phenylindole (DAPI) for 10 min and then visualized using a fluorescence microscope (Nikon Eclipse C1, Japan).

#### Western blotting (WB)

WB was performed to determine the expression of C3 (1:1000, EPR19394, Abcam), synapsin (SYN) (1:1000, EPR23531-50, Abcam), and postsynaptic density protein 95 (PSD95) (1:1000, EPR23124-118, Abcam) proteins in the hippocampus, hypothalamus, and PFC. A tissue protein extraction kit was used to extract total protein from the hippocampus, hypothalamus, and PFC of each mouse. A BCA protein assay (Beyotime) was performed using the protein extracts. The gel used for Western blotting in our study had a percentage of 10%. After electrophoresis and electroporation, the converted membrane was blocked with 5% skimmed milk powder for 1 h, washed with TBST buffer, and incubated with the primary antibody overnight at 4 °C. The electrophoretic membrane was incubated with a secondary antibody for 1 h, washed with TBST buffer, and luminescence induced using a chemiluminescence reagent (Millipore, Billerica, MA, USA). The protein separation membrane was scanned and analyzed using an image analyzer (Bio-Rad, Hercules, California, USA).

### Bacterial DNA extraction, 16S rRNA sequencing, and analyses

Microbial DNA was extracted from colonic contents using the E.Z.N.A.® DNA Kit (Omega Bio-Tek, Norcross, GA, U.S.), according to the manufacturer’s instructions. The final DNA concentration and purity were determined using a NanoDrop 2000 UV–vis spectrophotometer (Thermo Scientific, Wilmington, USA), and DNA quality was evaluated by 1% agarose gel electrophoresis.

The V3–V4 hypervariable regions of the bacterial 16S rRNA gene were amplified with primers 341F (5′-CCTAYGGGRBGCASCAG-3′) and 806R (5′-GGACTACNNGGGTATCTAAT-3′) using a thermocycler PCR system (GeneAmp 9700, ABI, USA). The PCR products were extracted from a 2% agarose gel using the AxyPrep DNA Gel Extraction Kit (Axygen Biosciences, Union City, CA, USA) and quantified using QuantiFluor™-ST (Promega, USA), according to the manufacturer’s instructions. Diversity metrics were calculated using the core-diversity plugin within QIIME2. Feature-level α-diversity indices, such as the observed Chao1 richness estimator and Shannon diversity index, were calculated to estimate microbial diversity within individual samples. The β-diversity distance measurements, including Bray–Curtis, unweighted UniFrac, and weighted UniFrac, were performed to determine the structural variation in microbial communities across samples and then visualized by principal coordinate analysis (PCoA). The relative abundances of microbial species at different taxa levels were estimated using the R package “vegan”. The sequencing and data analysis services were provided by Wekemo Tech Group Co., Ltd., Shenzhen, China.

### Metabolomic analyses

Metabolomics analyses were performed on microbial metabolite extracts using an ACQUITY UPLC I-Class PLUS/Xevo G2-XS QToF system (Waters Corporation, Milford, MA, USA) with a Waters Acquity UPLC HSS T3 column (2.1 × 100 mm, 1.8 μm). Samples (1 μL) were injected and eluted with a mobile phase comprising solvent A (0.1% formic acid) and B (0.1% formic acid-acetonitrile) at a flow rate of 0.4 mL/min and 40 °C column oven. The eluting gradient program was as follows: 0–0.25 min, 2.0% B; 0.25–10.00 min, 2.0–98% B; 10.00–13.00 min, 98% B; 13.0–13.10 min, 98–2.0% B; and 13.10–15.00 min, 2.0% B. The effluent was connected to an ESI-triple quadrupole linear ion trap (QQQ-LIT) mass spectrometer equipped with an ESI Turbo Ion-Spray interface operating in both positive and negative ion modes and operated using Analyst software. The ESI source operating parameters were as follows: ion source temperature, 150 °C; desolventizing gas temperature, 500 °C; capillary voltage, 2000 V; cone voltage, 30 V; cone-gas flow rate, 50 L/h; and desolventizing gas flow rate, 800 L/h. Full scans were acquired in a scan range of 50–1200 m/z with a scan frequency of 0.2 s, and data were collected by MassLynx (v. 4.2, Waters) and identified by Progenesis QI within a mass deviation of 100 ppm. The total metabolite concentration did not differ significantly between the samples. Principal component analysis (PCA) of the metabolites was performed using PAleontological STatistics software. Partial least squares-discriminant analysis (PLS-DA) was performed using SIMCA® software to improve visualization. The volcano map of total metabolites and mapping of differential metabolites via Kyoto Encyclopedia of Genes and Genomes (KEGG) enrichment analysis were drawn using the R package “ggplot2”. The PLS-DA model was assessed using Hotelling’s T2 test. The LAD score was evaluated using Kruskal–Wallis and Wilcoxon tests.

### Data correlation analysis

Microbial network analysis was performed using Spearman’s rank correlation analysis according to set conditions (correlation > 0.8; *P* < 0.05). Differential metabolite enrichment analysis was performed by using a hypergeometric test. Microbiome and metabolome correlation analyses were conducted using Pearson’s correlation; absolute values of CC > 0.8 and *P* < 0.05 were included. Statistical significance was set at *P* < 0.05. Gut microbiota, behaviors, and levels of IL-6, IL-10, IL-1β, and TNF-α in CUMS-induced depressed mice were evaluated by Pearson correlation analysis. All statistical analyses were performed using IBM SPSS (version 22), PAST (version 4.03), and SIMCA® (version 13.0). All graphs were constructed using GraphPad Prism 7 (La Jolla, CA, USA), PAST (version 4.03), SIMCA (version 13.0), Tbtools (version 1.049), Cytoscape (version 3.7.2), and R (version 4.0.2).

### Statistical analysis

Data are presented as the arithmetic mean ± standard error of the mean (SEM) using SPSS software (version 25.0; Chicago, IL, USA). Data produced by repeated measurements were first analyzed using repeated analysis of variance (ANOVA) if the data were normally distributed and homogenous. Besides, the post hoc test were conducted. If the data were not normally distributed or the variance was not uniform, a non-parametric test of K-independent samples was used for item-by-item statistical analysis. Statistical significance was set at *P* < 0.05. The graphs were constructed using GraphPad Prism 7.

## Results

### XYS, as potential antidepressant, alleviated CUMS-induced depression/anxiety-like behaviors, and cognitive impairment in a gut microbe-dependent manner

**Fig. 2:**
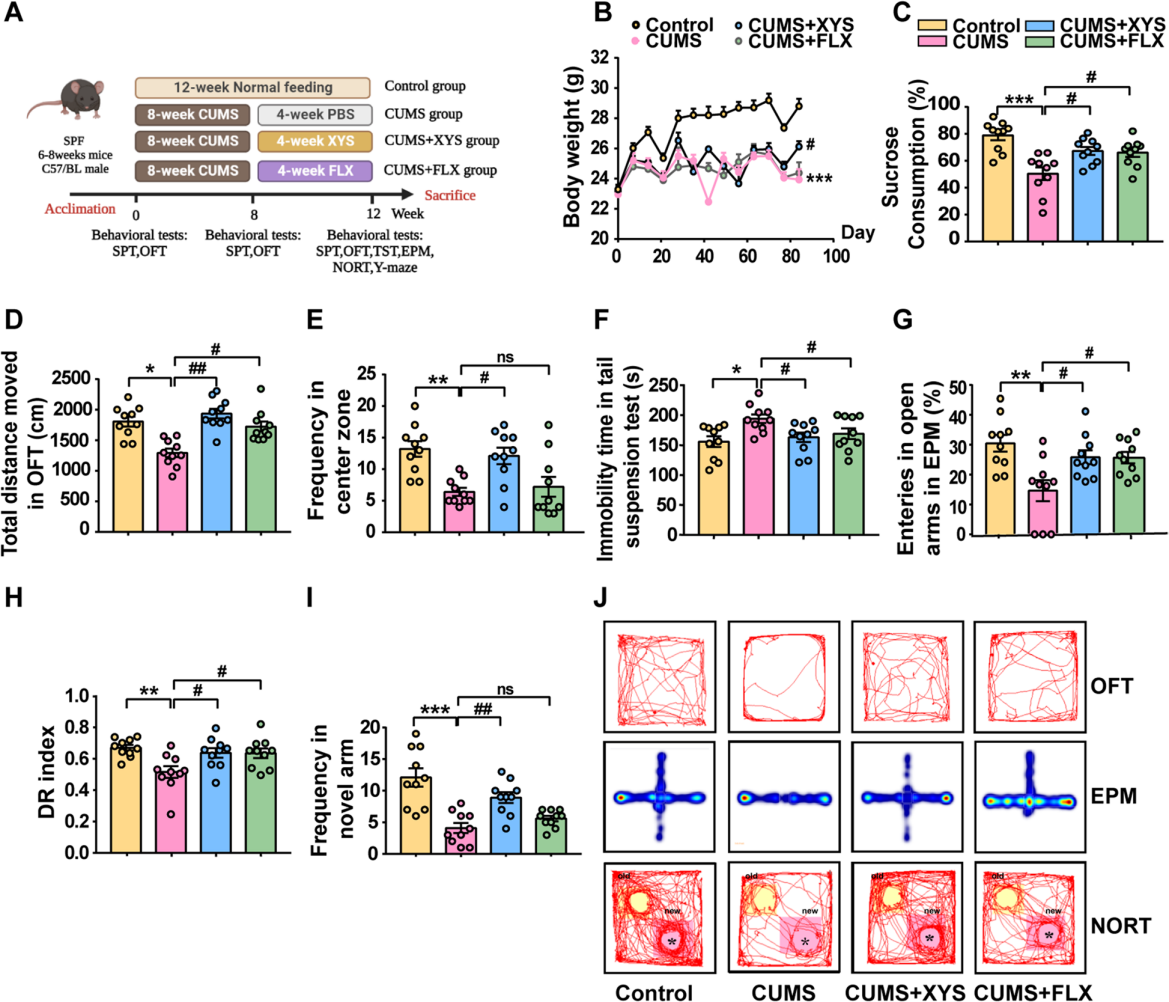
XYS, as potential antidepressant, alleviated CUMS-induced depression/anxiety -like behaviors, and cognitive impairment. **A** After 1 week of acclimation, mice were randomly divided into four groups as follows: Control, CUMS, CUMS + XYS, and CUMS + FLX. **B** Daily body weight changes throughout the entire duration of the study. **C** Sucrose preference test (SPT). **D** Open field test(OFT) (Total distance moved). **E** OFT (Frequency in center zone). **F** Tail suspension test (TST). **G** Elevated plus-maze (EPM). **H** Novel object recognition test (NORT). **I** Y-maze. **J** Movement trajectories of mice in OFT, EPM and NORT. Data represent the mean ± SEM (*n* = 10 per group). ^*^P < 0.05, ^**^P < 0.01, ^***^P < 0.001 versus the Control group; ^#^*P* < 0.05, ^##^*P* < 0.01 versus the CUMS group

The potential antidepressant activity of XYS on CUMS-induced depression-like behaviors was evaluated by inducing depression-like behaviors in mice by administering 8 weeks of CUMS, followed by 4 weeks of daily oral administration of XYS (Fig. 2A). No differences were observed in body weight, sucrose preference, or motor function among the groups before the experiment (Fig. S1a–c). Significant depression-like behaviors were observed in the mice that received the 8-week CUMS intervention (Fig. S1d–g). Following the successful establishment of the CUMS mouse model, the effects of XYS intervention on mouse behavior were examined. XYS intervention significantly alleviated CUMS-induced depression-like behaviors compared with the CUMS group, as evidenced by markedly reduced weight loss, increased preference in the SPT, increased total distance and entry frequency in the OFT and decreased immobility time in the TST (Fig. 2B–F). XYS treatment also significantly improved anxiety-like behaviors and cognitive impairment in mice with CUMS-induced depression-like behaviors. Mice receiving XYS intervention showed an increased frequency of open arm entry in the EPM (Fig. 2G) and increased exploration of novel areas in the NORT (Fig. 2H) and Y-maze tests (Fig. 2I). The movement trajectories of the mice in the OFT, EPM, and NORT are shown in Fig. 2J. The above results indicate that XYS has significant antidepressant effects and could be further studied as a potential antidepressant.

To further explore whether the destruction of gut microbiota affects the effectiveness of XYS in alleviating depression, an antibiotic cocktail was used to pretreat the mice before oral administration of XYS (Fig. S1h). The use of antibiotics to disrupt the intestinal flora can affect the improvement of depression-like behaviors by XYS in mice, indicating that the efficacy of XYS depends on the intestinal flora to a certain extent (Fig. S1i–j).

Collectively, these results indicate that CUMS can induce significant depression-like and anxiety-like behaviors and concomitant cognitive impairment in mice. Furthermore, depression symptoms were ameliorated by XYS, but pretreatment with antibiotics attenuated the antidepressant effect, suggesting that XYS alleviated CUMS-induced depression/anxiety-like behaviors and cognitive impairment in a gut microbe-dependent manner.

### Depression-like behaviors are accompanied by gut dysbiosis

Intestinal homeostasis is composed of the intestinal microecology and mucosal barrier (including intestinal mucosal immunity). To observe the changes in intestinal homeostasis during the depression, colonic inflammation and barrier function, intestinal microbial diversity, and microbial metabolites were determined.

**Fig. 3:**
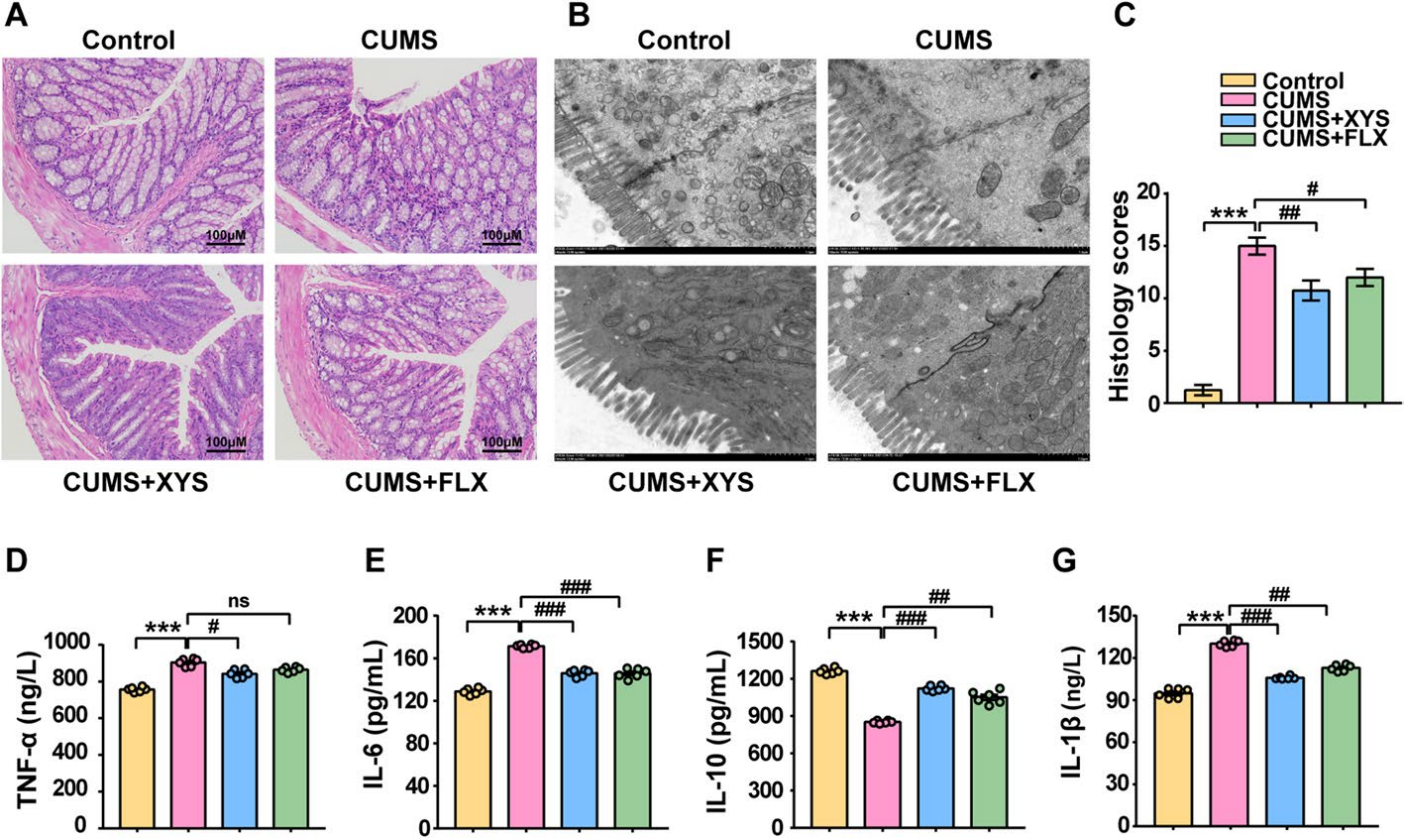
XYS suppressed CUMS-induced colonic inflammatory response, improved the barrier function. **A** H&E-stained colon sections (× 200). **B** Transmission electron microscopy (× 10,000, 1um). **C** Histological scores of colons. The level of **D** IL-1β; **E** TNF-α; **F** IL-6; **G** IL-10 in the serum. Data represent the mean ± SEM (*n* = 6 per group). ^***^P < 0.001 versus the Control group; ^#^*P* < 0.05, ^##^*P* < 0.01, ^###^*P* < 0.001 versus the CUMS group

To understand changes in colonic pathology, barrier function, and systemic inflammation during the development of depression, histological analysis, colon ultrastructural morphology, and the level of inflammatory cytokines in serum were evaluated. The mice with depression-like behaviors exhibited obvious colonic inflammation and significantly increased pathological scores compared with control mice (Fig. 3A, B). Transmission electron microscopy revealed a reduction in the size and number of microvilli and an abnormal appearance of the gut barrier in mice with depression-like behaviors (Fig. 3C). Aggravation of colonic inflammation and disruption of barrier function can affect the levels of peripheral inflammatory factors. IL-10 expression was inhibited in the CUMS model, and increased levels of IL-1β, IL-6, and TNF-α were observed in the sera of the CUMS mice with depression-like behaviors (Fig. 3D–G). Administration of antidepressants can improve colonic barrier function and inflammatory pathology. After XYS administration, intestinal inflammation was ameliorated, the size and number of colonic microvilli increased, and abnormal alignment was reversed (Fig. 3A–C). In addition, XYS reduced the levels of IL-1β, IL-6, and TNF-α and increased the level of IL-10 in the sera of the mice with depression-like behaviors (Fig. 3D–G).

**Fig. 4:**
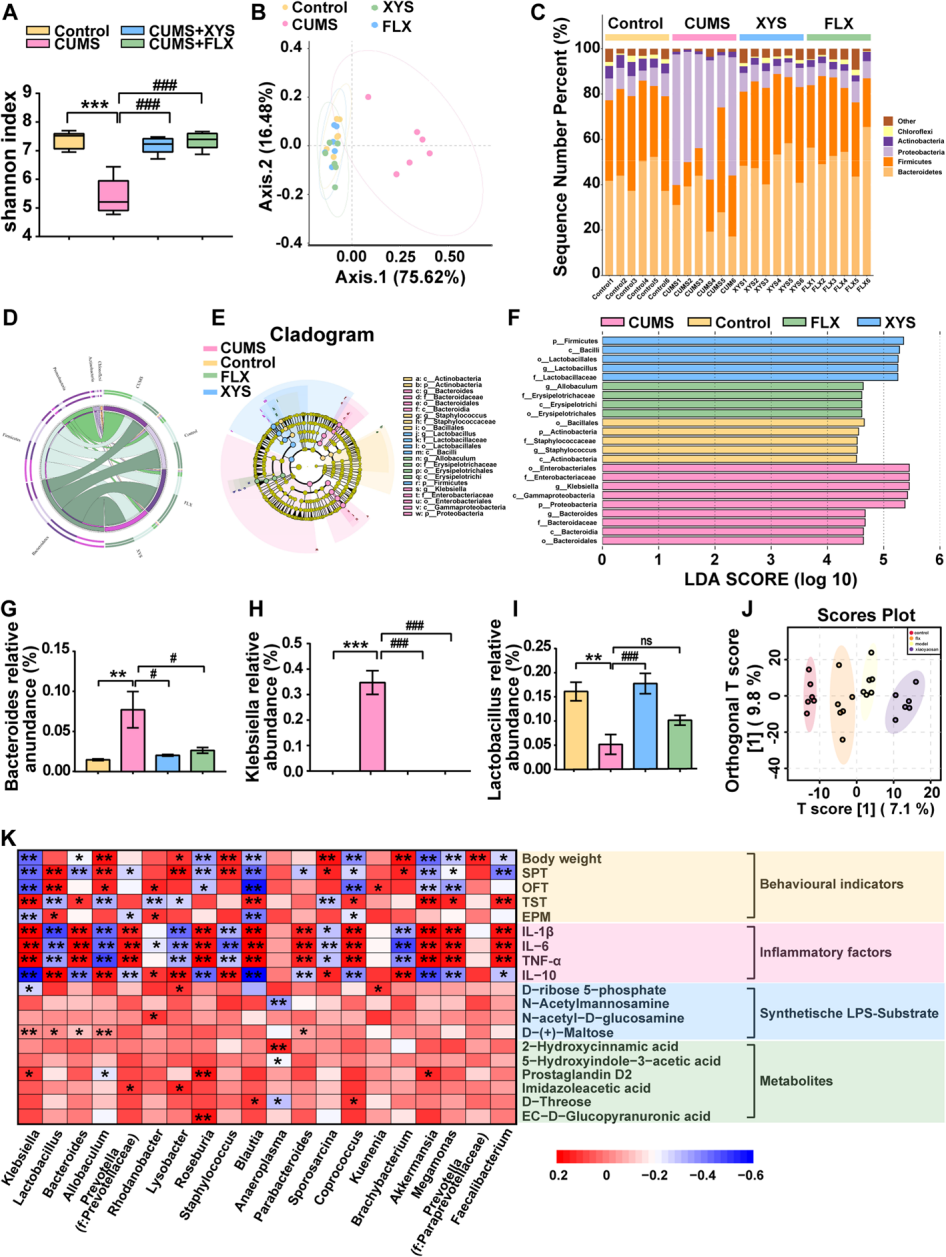
Depression-like behaviors are accompanied by dysbiosis in gut homeostasis, and antidepressants restore gut homeostasis. **A** Alpha diversity shown by Shannon index. **B** PCoA. **C** Gut microbiota change at phylum level (barplot). **D** Gut microbiota change at phylum level (circos). **E,F** Cladogram (Gut microbiota change at the family level). The relative abundances of G: *Bacteroides* spp.; **H** *Klebsiella* spp.; **I** *Lactobacillus.* **J** Changes in gut microbial metabolites(PLS-DA). **K** Spearman’s correlation analysis of microbiome and metabolome. Data represent the mean ± SEM (*n* = 6 per group). ^**^P < 0.01, ^***^P < 0.001 versus the Control group; ^#^*P* < 0.05, ^###^*P* < 0.001 versus the CUMS group

The changes in the gut microbiota and intestinal metabolites and the response to antidepressants in a depression model were evaluated via 16S rRNA gene sequencing and UHPLC-QTOF-MS/MS analysis. The α-diversity results shown by the Shannon index indicated that the gut microbial diversity was reduced in mice with depression-like behaviors and could be restored by oral antidepressants (XYS or FLX) (Fig. 4A; Fig. S2a–c: α-diversity shown by Chao, observed, and Simpson indices). Principal coordinate analysis (PCoA) based on the Bray–Curtis distance revealed a difference in the gut microbiota structure between control and mice with depression-like behaviors (Fig. 4B). A significant difference in the intestinal flora structure was observed between the CUMS + XYS and CUMS groups (*F* = 17.213, *P* = 0.001), indicating that the fecal microbiota structure in mice exhibiting depression-like behaviors was significantly affected by oral administration of XYS.

A total of 37 phyla were identified in this study. Firmicutes, Bacteroidetes, and Proteobacteria were the dominant phyla in the fecal microbiota of the mice. Proteobacteria was the dominant phylum in the fecal microbiota of mice with depression-like behaviors. XYS intervention significantly reduced the abundance of Proteobacteria compared with the CUMS mice without treatment (Fig. 4C, D; Fig. S2d). A cladogram generated by LEfSe analysis of the microbiome data (Fig. 4E, F) showed nine and five differentially abundant clades at the family level in the CUMS and XYS groups, respectively (*P* < 0.05, LDA > 4.0). At the genus level (Fig. 4G–I; Fig. S4e), the abundance of *Bacteroides* and *Klebsiella* was significantly increased in the CUMS group, while oral administration of XYS significantly decreased the relative abundance of *Bacteroides* and *Klebsiella* compared with the control group (*P* < 0.05 and *P* < 0.001, respectively). XYS administration significantly increased the relative abundance of *Lactobacillus* in the CUMS mice (*P* < 0.001).

**Fig. 5:**
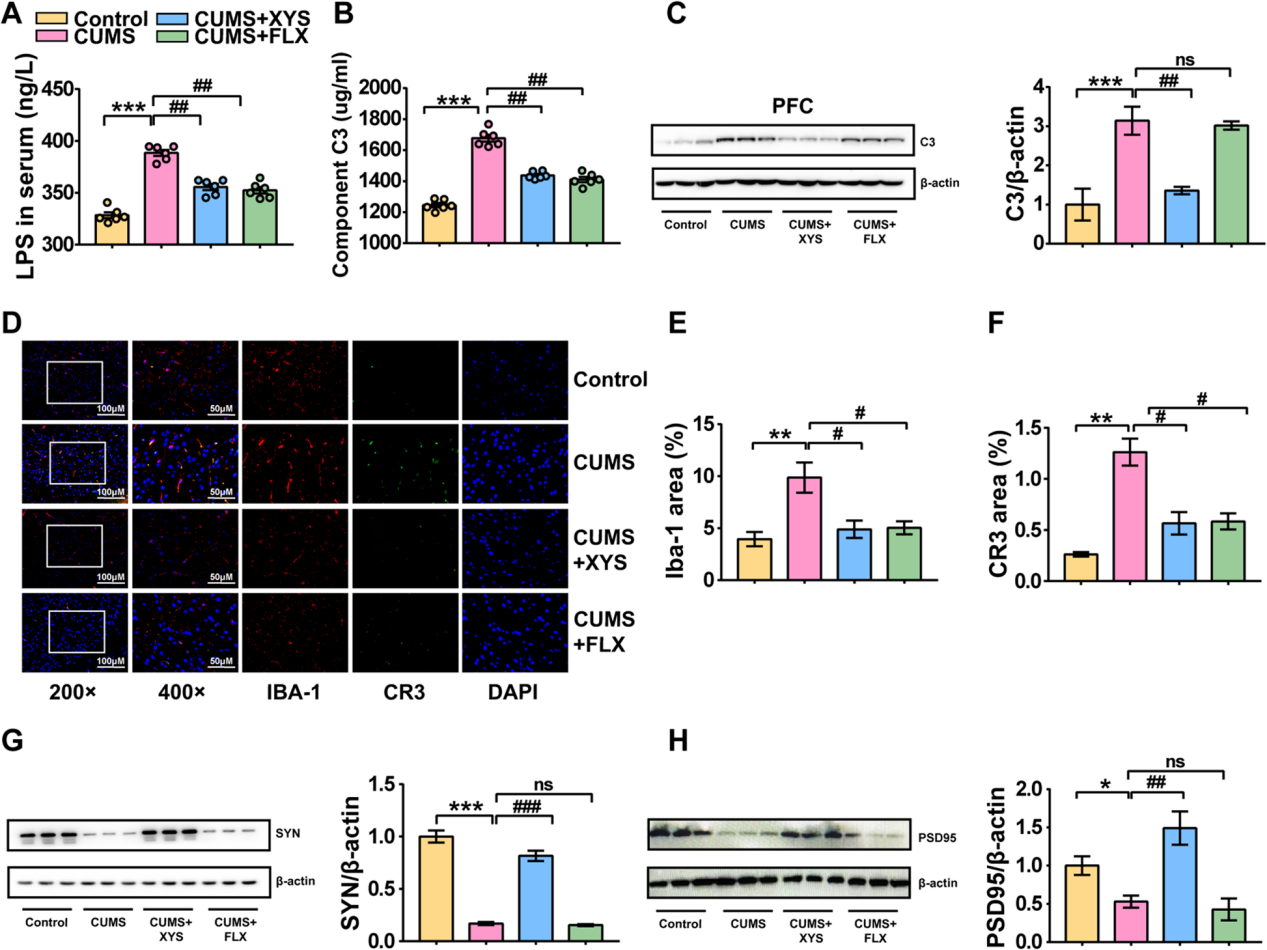
Complement C3/CR3 activation and complement-involved microglia-mediated aberrant synaptic pruning are important pathological manifestations of depression. **A** LPS in serum. **B** C3 in serum. **C** The protein expression level of C3 in PFC. **D** Immunofluorescence of microglia and CR3. **E** The expression of IBA-1 protein in PFC. **F** The expression of CR3 protein in PFC. **G** The protein expression level of SYN and PSD95 in PFC. Data represent the mean ± SEM (*n* = 6 per group for ELISA; *n* = 3 per group for IF and WB). ^**^P < 0.01, ^***^P < 0.001 versus the Control group; ^#^*P* < 0.05, ^###^*P* < 0.001 versus the CUMS group

To further explore whether changes in gut metabolites are involved in the formation of depression-like behaviors and regulation of antidepressants, the colonic contents of the mice were analyzed using UHPLC-QTOF-MS/MS in positive and negative modes. The PLS-DA method was applied to investigate the separation of the control, CUMS, CUMS + XYS, and CUMS + FLX groups. Separation was observed among the four groups, indicating metabolite changes in the development of depression-like behavior or ingestion of antidepressants (Fig. 4J). To further clarify these specific changes, a supervised multivariate orthogonal partial least squares-discriminant analysis (OPLS-DA) model was used to distinguish between the different variables. The CUMS group was separated from the control and CUMS + XYS groups (Fig. S2f–h). In addition, 2106 metabolites were identified using online databases. A total of 359 differential metabolites were identified in the comparison between the control and CUMS groups (*P* < 0.05), of which 219 were increased and 140 were decreased after CUMS induction. In addition, 240 different metabolites were identified in the comparison between the XYS and CUMS groups (*P* < 0.05), of which 79 were increased and 161 were decreased after XYS administration. KEGG topology analysis was used to identify pathways that were enriched between the groups (Fig. S2i, j). Among them, vitamin B6 metabolism (impact value > 5; *P* < 0.001) and lysine biosynthesis (impact value > 6; *P* < 0.001) showed significant differences (Fig. S2i, j). In addition, we performed Spearman’s correlation analysis of the microbiome and metabolites in colon content samples. Changes in the gut microbiota were most significantly associated with LPS biosubstrates (Fig. 4K). The concentration of LPS-produced substrates changed significantly as CUMS-induced gram-negative bacteria increased. Therefore, we focused on the concentrations of LPS-generated substrates (Fig. S2k). Two LPS-generated substrates (D-( +)-mannose and *N*-acetylmannosamine) were significantly diminished by CUMS, and two other LPS-generated substrates (D-ribose 5-phosphate and glucose-6-phosphate) were observed to decline (Fig. S2l–o). A decrease in the synthetic substrate indicates an increase in synthetic consumption. To verify the reliability of the metabolic detection, the LPS content in the serum was evaluated. The concentration of LPS in the serum of mice with depression-like behaviors was significantly increased compared with control mice (Fig. 5A). However, XYS administration attenuated LPS levels, which is consistent with the sequencing results observed in the metabolomics analyses.

Collectively, these results indicate that the development of depression-like behavior is accompanied by dysbiosis in gut homeostasis (including colonic inflammatory response, destruction of colonic barrier function, reduction in intestinal microbial diversity, and accumulation of microbial metabolite-LPS). However, antidepressants suppress the CUMS‑induced colonic inflammatory response, improve barrier function and regulate the composition and metabolism of gut microbiota (especially the inhibition of gram-negative bacteria abundance and levels of LPS).

### Complement C3/CR3 activation and complement-involved microglia-mediated aberrant synaptic pruning are important pathological manifestations of depression

The occurrence of depression-like behavior was accompanied by disturbances in the composition, structure, and metabolism of the gut microbiota and XYS was able to restore gut homeostasis. However, whether complement system-mediated central synaptic pruning is involved in the emergence of depression-like behavior and regulation of complement by antidepressants remains unclear. As an important pathway for complement system activation, LPS was shown to be highly expressed in the sera of mice with depression-like behaviors (Fig. 5A). Possible changes in the peripheral and central complement systems and synaptic pruning were explored. A significant elevation in complement C3 levels was observed in serum samples (Fig. 5B). In addition, the expression of C3 in the CNS was measured. The protein expression levels of complement C3 in the hippocampus, hypothalamus, and prefrontal cortex were evaluated. Complement C3 expression was significantly increased in the prefrontal cortex (Fig. 5C). The complement system is involved in the regulation of microglial synaptic pruning in the brain. Complement C3 induces microglia to engulf labeled synapses by directing the growth of chemotactic microglia and binding to the complement receptor on microglia. To evaluate synaptic pruning in the central microglia, the changes in LPS-induced CD68 and IBA-1 expression levels were determined. The immunofluorescence results indicated that CUMS-induced PFC microglia exhibited specific activation of LPS (Fig. S3a, b). Besides, the size of the microglial soma was increased in CUMS-induced mice with a more ramified phenotype (shown in Additional file 3). In addition, the expression and localization of complement C3 receptor-CR3 in the PFC were observed using immunofluorescence. CUMS induced an increase in CR3 expression in the PFC. This suggests that the activation of C3/CR3 signaling is co-localized with microglia in the cytoplasm during the development of depression (Fig. 5D–F). The synaptic proteins SYN and PSD95 are important indicators that reflect the growth and maintenance of synapses. The protein expression of SYN and PSD95 was significantly decreased in the prefrontal cortex of mice subjected to CUMS, suggesting impaired synaptic function during the depression (Fig. 5G). Meanwhile, XYS administration reduced the expression of complement C3 in the peripheral and prefrontal cortices, inhibited the activation of the C3/CR3 pathway, and increased the expression levels of synaptic proteins SYN and PSD95, indicating that antidepressants suppress complement-involved microglia-mediated abnormal synaptic pruning, maintaining the normal growth of synaptic neurons.

Collectively, these results indicate that complement C3/CR3 activation and microglia-mediated aberrant synaptic pruning are important pathological manifestations of depression. Meanwhile, antidepressants suppress the CUMS‑induced activation of complement C3/CR3 and complement-involved microglia-mediated abnormal synaptic pruning, thereby maintaining the normal growth of synaptic neurons.

### Transplantation of dysregulated gut microbiota induces the development of depression-like behaviors

**Fig. 6:**
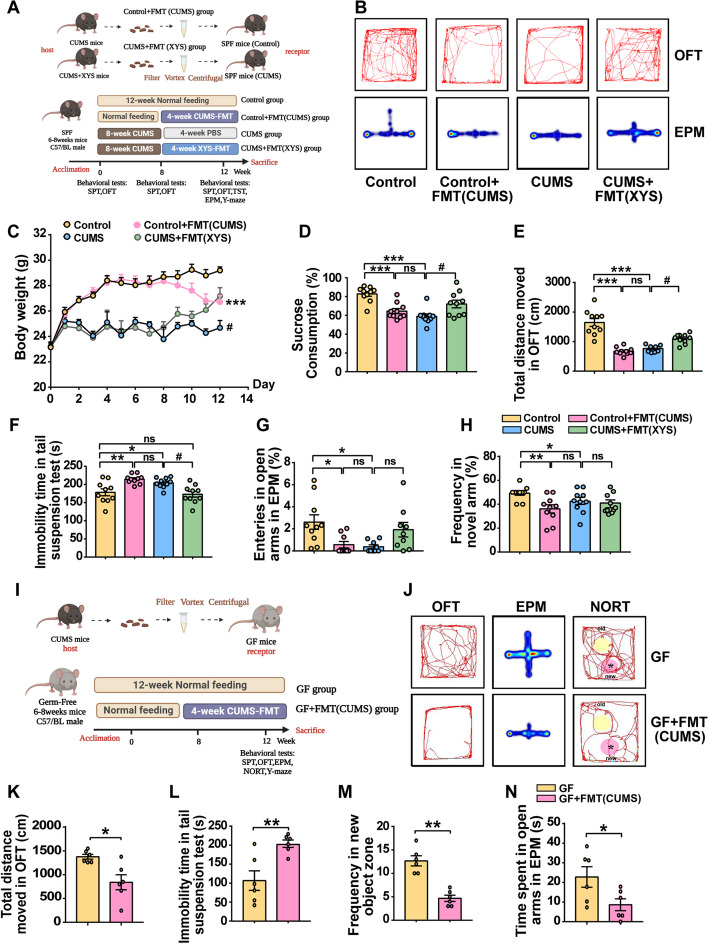
Transplantation of dysregulated gut microbiota directly induces development of depression-like behaviors. **A** After 1 week of acclimation, mice were randomly divided into four groups as follows: Control, Control + FMT(CUMS), CUMS and CUMS + FMT(XYS). **B** Body weight (SPF mice). **C** SPT (SPF mice). **D** OFT (Total distance moved) (SPF mice). **E** TST (SPF mice). **F** EPM (SPF mice). **G** Y-maze (SPF mice). **H** Movement trajectories of mice in OFT, EPM (SPF mice). **I** Study design for the GF mice. **J** OFT (GF mice). **K** TST (GF mice). **L** NORT (GF mice). **M** EPM (GF mice). **N** Movement trajectories of mice in OFT, EPM (GF mice). Data represent the mean ± SEM (*n* = 10 per group for SPF mice; *n* = 6 per group for GF mice). ^*^P < 0.05, ^**^P < 0.01, ^***^P < 0.001 versus the Control group; ^#^*P* < 0.05, ^##^*P* < 0.01 versus the CUMS group

The connection between gut microbiota and the onset of depression was verified. The impact of CUMS-mediated microbiota on SPF and GF mice was determined by transplanting fecal microbiota derived from CUMS-induced mice (Fig. 6A). Animal behavior was evaluated before FMT (Fig. S4a–f). Both SPF and GF mice exhibited significant depression-like and anxiety-like behaviors after FMT, which were accompanied by impairments in cognitive memory. SPF mice receiving the fecal microbiota of CUMS-induced mice exhibited weight loss, decreased sucrose preference, decreased total distance in the OFT, and decreased desire for novelty in the NORT and Y-maze compared with the control mice; this is similar to mice after 8-week CUMS stimulation (Fig. 6B–H). Similar results were observed in GF mice. GF mice receiving the fecal microbiota of CUMS mice showed a significant reduction in total movement distance in the OFT, increased immobility time in the TST (Fig. 6I–K), and cognitive impairment in the NORT and Y-maze (Fig. 6L–N). These results suggest that under stress-independent conditions, receiving a FMT from a CUMS host with depressive-like behavior and cognitive impairment can lead to behavioral abnormalities, including depression-like behaviors.

Furthermore, the impact of antidepressant-mediated microbiota on depression was evaluated by transplanting fecal microbiota derived from mice administered XYS into CUMS-induced mice. CUMS mice receiving fecal microbiota from XYS-treated mice had significantly improved weight gain and sucrose preference compared with CUMS-induced mice without treatment (Fig. 6B–E). However, improvements in anxiety-like behavior and cognitive impairment were not observed in mice receiving fecal microbiota derived from XYS-treated mice (Fig. 6F,G). The associated behavioral changes suggested a trend toward remission without statistical significance.

These results indicate that CUMS-FMT contributed to depressive-like behavior, anxiety-like behavior, and cognitive impairment, while XYS-FMT contributed to depression alleviation, but not alleviation of anxiety-like behavior and cognitive impairment. This suggests that gut dysbiosis induces the development of depression-like behaviors, whereas FMT treated with antidepressants has an inhibitory effect on depression-like behaviors.

### Dysregulated gut microbiota mediated colonic inflammation and barrier disruption

**Fig. 7:**
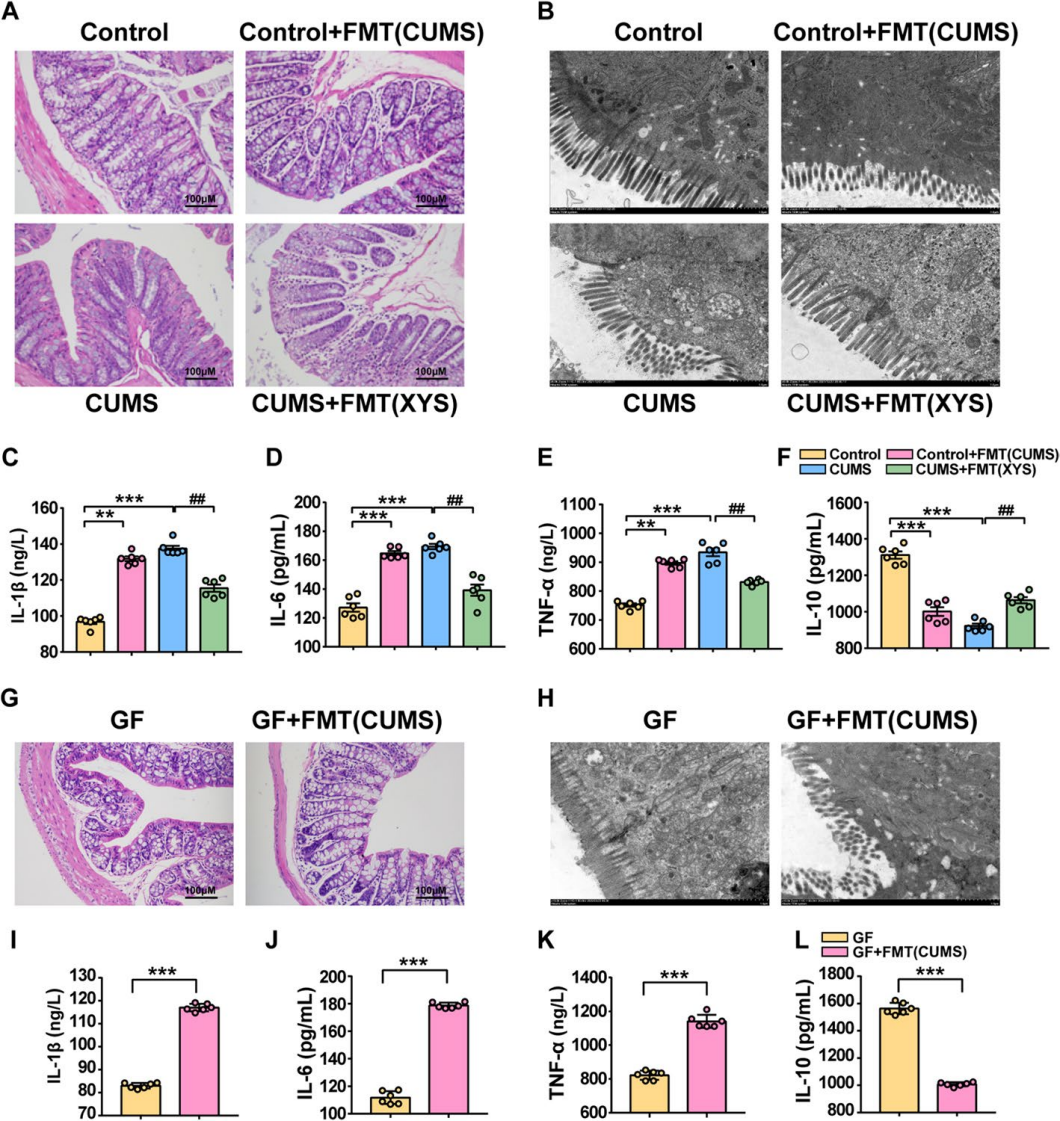
CUMS-FMT contributed to colonic inflammatory response, intestinal barrier damage; XYS-FMT suppressed CUMS-induced colonic inflammatory response, improved the barrier function. **A** HE (SPF mice). **B** Transmission electron microscopy (SPF mice). **C** IL-1β (SPF mice). **D** IL-6 (SPF mice). **E** TNF-α (SPF mice). **F** IL-10 (SPF mice). **G** HE (GF mice). **H** Transmission electron microscopy (GF mice). **I** IL-1β (GF mice). **J** IL-6 (GF mice). **K** TNF-α (GF mice). **L** IL-10 (GF mice). Data represent the mean ± SEM (*n* = 6 per group). ^**^P < 0.01, ^***^P < 0.001 versus the Control group; ^#^*P* < 0.05, ^##^*P* < 0.01 versus the CUMS group

FMTs from the CUMS group to control mice and the XYS + CUMS group to CUMS-induced mice were performed to investigate the bioactivities of CUMS-FMT and XYS-FMT on colonic inflammation and intestinal barrier function. HE staining, colon ultrastructural morphology, and serum levels of IL-1β, IL-6, TNF-α, and IL-10 were measured. Both GF and SPF mice receiving fecal microbiota from CUMS-induced mice exhibited significant colonic inflammatory syndromes compared with control mice (Fig. 7A). Transmission electron microscopy showed that the microvilli were reduced in size and number and exhibited an abnormal appearance (Fig. 7B). Decreased concentrations of IL-10 and increased levels of IL-1β, IL-6, and TNF-α were also observed in SPF and GF mice receiving fecal microbiota from CUMS-induced mice (Fig. 7C–F, G,H).

The impact of antidepressant-mediated microbiota on depression was validated by transplanting fecal microbiota derived from mice administered XYS into CUMS-induced mice. In SPF mice, the CUMS-induced colonic inflammation was reversed by XYS-FMT (Fig. 7A). Transmission electron microscopy showed that the microvilli were reduced in size and number and exhibited an abnormal appearance in the CUMS group. The size and number of microvilli increased and the abnormal arrangement was reversed by XYS-FMT (Fig. 7B). XYS-FMT increased the levels of IL-10 and inhibited IL-1β, IL-6, and TNF-α expression compared with that of the CUMS group (Fig. 7C–F).

These results indicate that CUMS-FMT contributes to colonic inflammatory response and intestinal barrier damage. In addition, XYS-FMT suppressed the CUMS‑induced colonic inflammatory response and improved the barrier function. This suggests that dysregulated gut microbiota mediates colonic inflammation and barrier disruption, while FMT treatment with antidepressants has an inhibitory effect on colonic inflammation and barrier disruption.

### CUMS-FMT mediated the disturbance of gut microbiota and XYS-FMT restored the intestinal balance

**Fig. 8:**
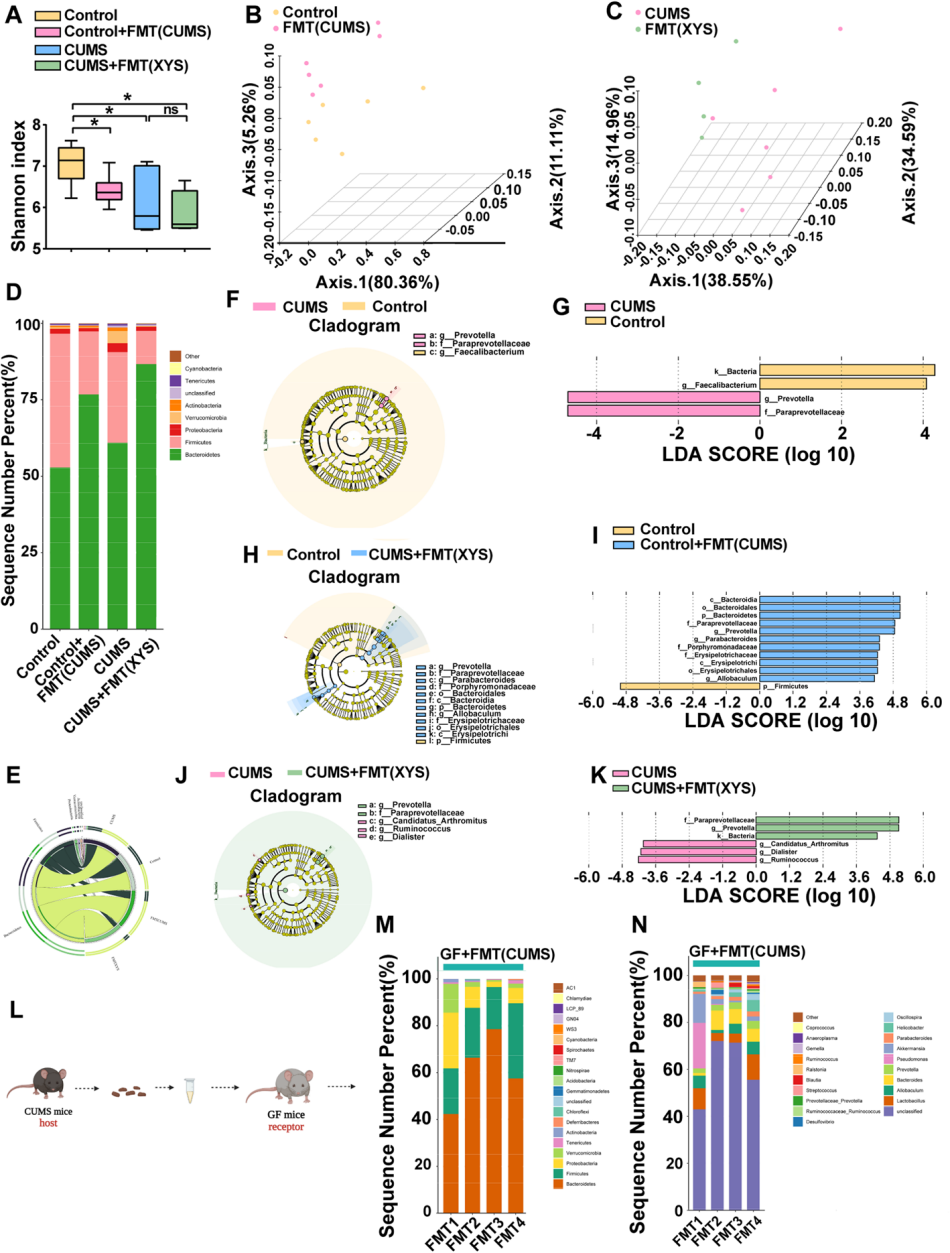
CUMS-FMT mediated disturbance of the gut microbiota in mice; XYS-FMT regulated the composition of gut microbiota. **A** Alpha diversity shown by Shannon index. **B** PCoA (Control versus Control + FMT(CUMS)). **C** PCoA (CUMS versus CUMS + FMT(XYS)). **D** Gut microbiota change at phylum level (barplot). **E** Gut microbiota change at phylum level (circos). **F,G** Cladogram (Control versus CUMS)). **H,I** (Control versus Control + FMT(CUMS)). **J,K** Cladogram (CUMS versus CUMS + FMT(XYS)). **L** Gut microbiota change at phylum and genus level of GF after FMT. Data represent the mean ± SEM (*n* = 6 per group). ^**^P < 0.01, ^***^P < 0.001 versus the Control group; ^#^*P* < 0.05, ^###^*P* < 0.001 versus the CUMS group

The impact of CUMS-FMT and XYS-FMT on the gut microbiota composition of mice was further explored using 16S rRNA gene sequencing. The α-diversity, shown by the Shannon index, was impacted by FMT (Fig. 8A; Fig. S5a–c: α-diversity shown by Simpson, observed, and Chao indices). PCoA based on the Bray–Curtis distance showed a separation in the gut microbiota structure among control mice with or without CUMS-FMT (Fig. 8B). A separation in the gut microbiota structure was also observed between the CUMS and CUMS + FMT (XYS) groups, which indicated that the gut microbial structure in mice with depression was affected by XYS-FMT (Fig. 8C).

In addition, the effects of stress and FMT on the gut microbial structure of mice were explored. First, our experimental results replicated the regulation of gut microbiota by stress in Experiment 1. This was consistent with the results shown in Fig. 8 D and E; the abundance of Proteobacteria in mice subjected to CUMS increased at the phylum level compared with that in the control group, indicating the consistency of changes in the microbiota under the same stress conditions. Additionally, an increased abundance of the phylum Verrucomicrobia was observed. A cladogram was generated by LEfSe analysis of microbiome data (Fig. 8F, G) and showed two differentially abundant clades at the genus level in the CUMS group, and two differentially abundant clades in the control group (*P* < 0.05, LDA > 4.0). At the genus level, CUMS mice showed a significant decrease in the abundance of *Lactobacillus* and the significant increase in the abundance of *Prevotella* (Prevotellaceae) compared with that of the control group (Fig. S5d).

Next, the effects of FMT on the host gut microbiota were assessed. Previous results have indicated that SPF mice receiving CUMS-FMT exhibit pronounced depression-like behaviors. Correspondingly, SPF mice receiving CUMS-FMT also exhibited an altered gut microbial structure and composition. At the phylum level, the abundance of Bacteroidetes was significantly increased in mice receiving CUMS-FMT compared with that of the control group, while there was no significant difference in the abundance of *Proteobacteria* (Fig. 8D, E). However, at the genus level, a significant effect was induced by FMT. A cladogram was generated by LEfSe analysis of microbiome data (Fig. 8H, I) and showed 11 differentially abundant clades at the genus level in the CUMS-FMT group and one differentially abundant clade at the genus level in the control group (*P* < 0.05, LDA > 4.0). Furthermore, at the genus level, the abundance of *Lactobacillus* and *Akkermansia* was significantly reduced in the gut microbiota of mice after CUMS-FMT treatment (Fig. S5e). This suggests that in SPF mice, FMT may not reproduce the host microbial signature due to the subject’s own gut microbial resistance, but rather modulate the abundance of specific genera in the form of microbial perturbations that induce gut microbial imbalances.

The effect of XYS-FMT was assessed in CUMS mice. Previous experiments have shown that XYS administration and XYS-FMT can modulate depression-like behavior in CUMS mice. Correspondingly, CUMS mice receiving XYS-FMT also exhibited an altered gut microbial structure and composition. At the phylum level, the abundance of Proteobacteria was significantly decreased in mice receiving XYS-FMT compared to that in the CUMS group, which is consistent with the changes following XYS ingestion (Fig. 8D, E). A cladogram was generated by LEfSe analysis of microbiome data (Fig. 8J, K) and showed three differentially abundant clades at the genus level in the XYS-FMT group, and three differentially abundant clades at the genus level in the CUMS group (*P* < 0.05, LDA > 4.0). Furthermore, at the genus level, the abundance of *Lactobacillus* and *Akkermansia* was significantly increased in the gut microbiota of mice after XYS-FMT (Fig. S5f).

Additionally, changes in the microbiota of GF mice that received CUMS-FMT were observed. GF mice do not harbor any known microbes in their intestines; therefore, the changes in their microbiota were more pronounced than those of SPF mice. At the phylum level, Proteobacteria was one of the dominant phyla in mice following CUMS-FMT, similar to the microbiota of mice subjected to CUMS. Consistently, at the genus level, the abundance of *Bacteroides* corresponded to the microbiota of mice exposed to stress (Fig. 8L).

### Dysregulated gut microbiota-induced complement C3 activation and microglia-mediated aberrant synaptic pruning

**Fig. 9:**
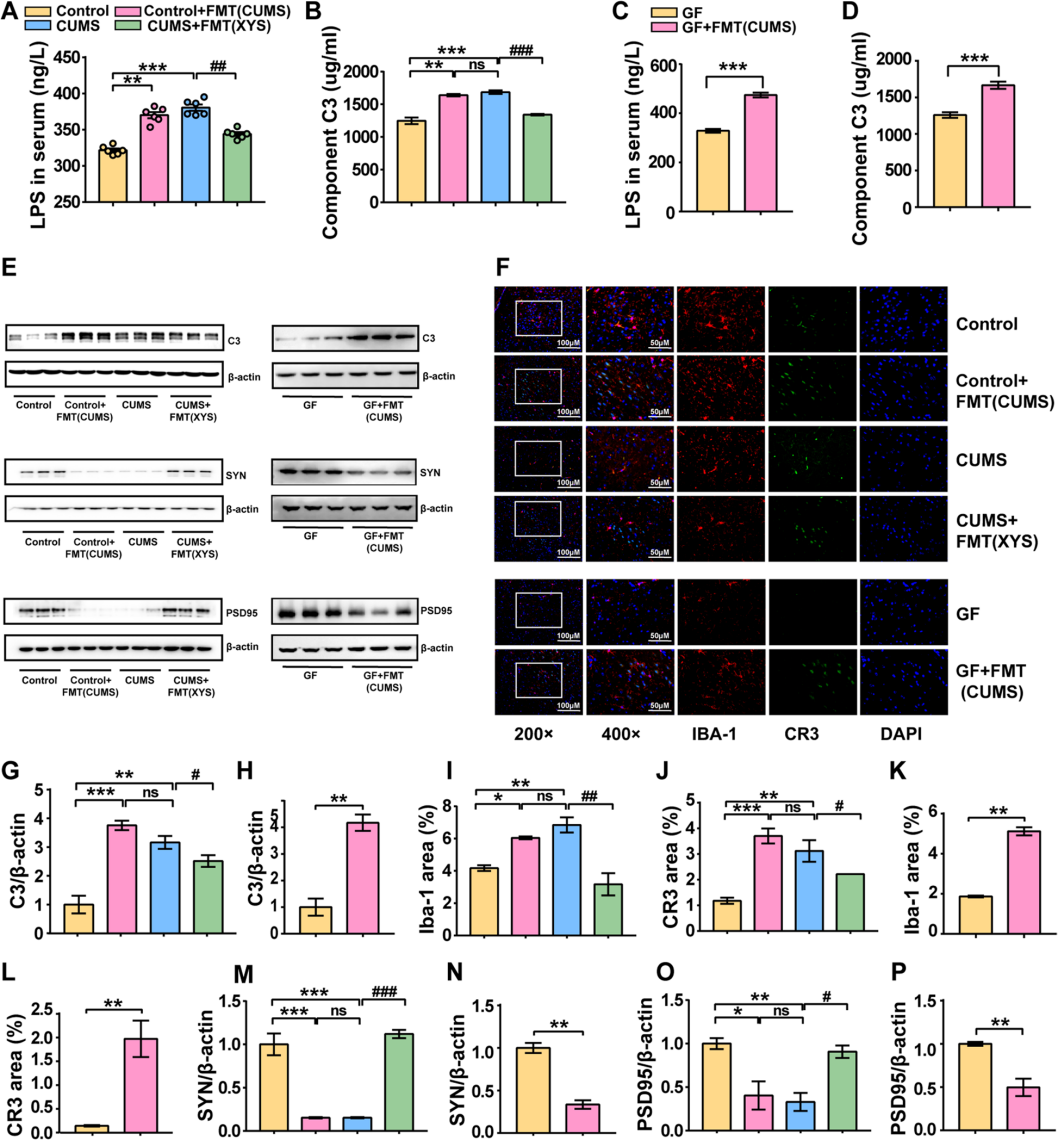
Dysregulated gut microbiota induced complement C3 activation and microglia-mediated aberrant synaptic pruning. **A** LPS in serum (SPF mice). **B** C3 in serum (SPF mice). **C** LPS in serum (GF mice). **D** C3 in serum (GF mice). **E** WB. **F** Immunofluorescence of microglia and CR3. **G** The expression of C3 protein (SPF mice). **F** The expression of C3 protein (GF mice). **I** The expression of IBA-1 protein (SPF mice). **J** The expression of CR3 protein (SPF mice). **K** The expression of IBA-1 protein (GF mice). **L** The expression of CR3 protein (GF mice). **M** The protein expression level of SYN (SPF mice). **N** The protein expression level of SYN (GF mice). **O** The protein expression level of PSD95 (SPF mice). **P** The protein expression level of PSD95 (GF mice). Data represent the mean ± SEM (*n* = 6 per group for ELISA; *n* = 3 per group for IF and WB). ^**^P < 0.01, ^***^P < 0.001 versus the Control group; ^#^*P* < 0.05, ^###^*P* < 0.001 versus the CUMS group

The emergence of depression-like behavior and the mechanism of antidepressants involves changes in the gut microbiota, complement C3/CR3, and synaptic pruning. However, it is unclear whether the activation of complement C3/CR3 in the PFC and microglia-mediated aberrant synaptic pruning are directly associated with changes in gut microbiota. To explore whether fecal microbial colonization without stressful stimulation could induce alterations in complement C3 and aberrant microglia-mediated synaptic pruning, FMT was performed from CUMS-induced mice into SPF and GF mice. The LPS and C3 levels in mice sera were significantly increased after receiving the fecal microbiota of CUMS-induced mice compared with that of control SPF mice (Fig. 9A, B). Consistent results were also reported for GF mice (Fig. 9C, D). Furthermore, consistent with CUMS induction, significantly increased protein expression of complement C3 was observed in the PFC tissues of both SPF and GF mice after receiving the fecal microbiota of CUMS-induced mice (Fig. 9E). In addition, immunofluorescence results based on CR3 and IBA-1 demonstrated that CUMS-FMT induced the activation of microglia in the PFC and increased CR3 expression, suggesting that CUMS-FMT could directly induce the activation of the C3/CR3 pathway (Fig. 9F–L). LPS-induced changes in CD68 and IBA-1 expression levels were also consistent with CUMS-induced stress (Fig. S3d–i). Decreased expression levels of SYN and PSD95 were observed, which also indicated neuronal damage caused by abnormal synaptic pruning (Fig. 9M–P; Additional file 3).

We also assessed the effect of antidepressant-FMT on synaptic pruning in CUMS mice. Our research showed that XYS administration can modulate C3-mediated aberrant synaptic pruning in CUMS mice. Correspondingly, XYS-FMT significantly reduced the levels of LPS and C3 in the sera of CUMS mice, inhibited the activation of microglia and the C3/CR3 pathway in the PFC, and increased SYN and PSD95 expression levels. These results suggest that gut microbiota intervention with XYS is of great significance for its antidepressant effects (Fig. 9A–P). The immunofluorescence results based on CD68 and IBA-1 indicated that the CUMS-induced activation of microglia in the PFC could be reversed by XYS-FMT (Fig. S3d, e; Additional file 3).

Collectively, these results indicate that dysregulated gut microbiota mediates complement C3 activation and microglia-mediated abnormal synaptic pruning, whereas FMT treatment with antidepressants suppressed CUMS-induced activation of complement C3 and complement-involved microglia-mediated abnormal synaptic pruning.

## Discussion

In the present study, we demonstrated that gut microbiota dysbiosis induces C3/CR3 system activation to promote synaptic pruning and precipitate depression-like behaviors. Evidence is presented that the administration of an antidepressant (XYS) ameliorated depression-like behaviors, anxiety-like behaviors, and cognitive impairment in mice as well as prevented gut microbiota imbalance-induced complement disturbances and abnormalities in complement-mediated synaptic pruning. Furthermore, CUMS-FMT in SPF and GF mice confirmed a direct association between gut microbiota, the complement system, and microglial synapse pruning. XYS-FMT significantly improved depressive-like behaviors in mice, suggesting that the underlying mechanisms of XYS-antidepressant efficacy mainly rely on regulating the interaction between the gut and brain. In addition, using broad-spectrum antibiotics nullifies antidepressant-induced behavioral improvement, which further highlights the essential role of the gut microbiota in mediating depression-like behaviors.

The microbiota-gut-brain axis is considered a key regulator of neural function and plays a key role in the pathogenesis of depression [30, 31]. Manipulation of gut microbes is considered a potential treatment for depression [32]. As a botanical formulation widely used in Asia and Europe, XYS contains a variety of active substances and is considered to have prebiotic-like properties [20]. In the current study, XYS, a potential antidepressant drug, was used to explore the role of gut microbiota in the treatment of depression and the specific mechanisms underlying the interaction between gut microbes and the CNS. Behavioral tests including SPT, OFT, TST, EPM, NORT, and Y-maze tests were used to explore the improvement effect of XYS on depression and its accompanying symptoms, such as anxiety-like behavior and cognitive impairment, to confirm its potential as an antidepressant. In addition, using 16S RNA sequencing and UHPLC-QTOF-MS/MS metabolic analysis, the improvement in depressive-like behaviors was shown to be accompanied by the regulation of gut microbes and microbial metabolites. Next, to verify whether the prebiotic-like properties of antidepressants play a key role in the improvement of depressive-like behaviors, ABX and FMT interventions were used to explore the antidepressant effect of XYS in different gut microbial environments. A variety of antibiotics were mixed into the daily drinking water of the mice and mice were allowed to drink freely for 7 days. The abovementioned antibiotic intervention method was chosen because previous studies have shown that intraperitoneal injection of antibiotics or long-term intervention (≥ 2 weeks) with mixed antibiotics can lead to depressive-like behaviors in mice [33, 34]. Besides, our findings suggested that 1 week of oral antibiotic treatment does not impact SPF behavior (The study results were shown in additional file 4). Therefore, to avoid the effect of antibiotic intervention on the depressive state of mice, a short-term (7 days) antibiotic intervention was administered to eliminate and manipulate the gut microbes of mice without aggravating the depressive-like behavior of mice. The antidepressant effect of XYS was eliminated after administering ABX for gut microbiota clearance, suggesting that the presence of gut microbes is necessary for XYS to exert its antidepressant effect. On the one hand, the disappearance of the microbiome results in the absorption and metabolism of the antidepressant active substances in XYS being inhibited and the subsequent antidepressant activities being ineffective; on the other hand, due to the changes in the intestinal microenvironment, the prebiotic-like effects of XYS associated with anti-depression cannot be exerted. Therefore, the FMT method was used to explore the improvement of depression-like behavior in mice treated with XYS-treated host fecal bacteria (gut microbiota, with or without metabolites). Our results indicate that transplanting feces from the antidepressant-treated host into depressed mice ameliorated depression-like behaviors in mice subjected to CUMS. This suggests that XYS can directly ameliorate depression symptoms through the colonization of the intestinal flora. These results confirm that the pharmacodynamic action of XYS as a potential antidepressant is mainly based on its prebiotic-like properties.

An imbalance of the intestinal microbiome is believed to drive the pathogenesis of depression. The results from 16 observational and 11 clinical studies confirmed that patients with major depression-like disorders exhibit changes in the intestinal microbiome [35]. Similar results were observed in rodents with depression-like behavior [36]. After confirming that the prebiotic-like properties of XYS are critical to improving depression-like behaviors, the specific changes in gut microbiota and microbial metabolites were explored. In the current study, XYS administration ameliorated the shift in the gut microbiota composition induced by CUMS. Microbiome studies using 16S rRNA gene sequencing have demonstrated that the abundance of the phylum Proteobacteria in the XYS group was significantly decreased, while the abundance of the phylum Bacteroidetes was increased compared with that in CUMS-induced mice. Proteobacteria is a microbial signature of dysbiosis in the gut microbiota [37]. Generally, commensal bacteria of Proteobacteria are present in the gut of healthy mammals. If these bacteria are present in low numbers, they appear benign; if their abundance is significantly elevated, they become inflammation-inducing gut microbes [38]. Extensive evidence has revealed that elevated Proteobacteria is a risk factor for psychiatric disorders such as depression and anxiety [39]. Antibiotic exposure severely damages the gut microbiota and significantly increases the abundance of Proteobacteria, leading to anxiety and depression [40]. Consistently, in the present study, the abundance of Proteobacteria was significantly elevated in the CUMS-induced depression model. The LDA analysis showed that XYS supplementation decreased not only the Proteobacteria at the phylum level, but also its lower taxa, such as Enterobacteriales, *Enterobacteriaceae*, and *Klebsiella* at the order, family, genus levels, respectively, in the CUMS-induced mice. Previous studies have shown that oral gavages of *Klebsiella oxytoca*, *Escherichia coli*, and *Cronobacter sakazakii* belonging to Enterobacteriaceae, in isolation or combination, cause depression-like behaviors in both GF and SPF mice, suggesting that Proteobacteria are closely associated with depression [41]. The microbiota belonging to the phylum Bacteroidetes has been associated with cognition and neurodegenerative diseases. Clinical studies have shown that infants with high levels of Bacteroides in the gut exhibit higher cognitive performance at 1 and 2 years of age [42]. In the present study, we found that XYS increased the abundance of Bacteroidetes at the phylum level, suggesting that it may be responsible for improving cognitive impairment. Furthermore, at the genus level, XYS decreased the abundance of *Bacteroides* and increased the abundance of *Lactobacillus*. *Bacteroides* are thought to be associated with major depressive disorder (MDD) [43]. High levels of *Bacteroides* spp. were present in fecal samples from 156 patients with MDD, compared with healthy individuals [44]. *Lactobacillus* is regarded as a probiotic with an inhibitory effect on depression [45]. It has been reported that the production of hydrogen peroxide by *Lactobacillus* may prevent CUMS-induced depressive behaviors by directly inhibiting intestinal indoleamine-2,3-dioxygenase [46]. We found that XYS administration significantly reduced the expression of *Bacteroides* and increased the expression of *Lactobacillus*. Overall, these findings support that XYS supplementation improves gut microbiota composition, particularly for the suppression of Proteobacteria and promotion of Bacteroidetes, which may contribute to the prevention of depression-like behaviors and concomitant cognitive impairment in mice with chronic stress-induced depression. In addition, the diversity of fecal microbiota is lower in patients with depression than in healthy controls. The gut microbiota diversity was reduced in CUMS mice, and XYS supplementation prevented these alterations in the gut microbiota. Therefore, it is speculated that the regulation of antidepressants on the composition and diversity of the gut microbiota may be crucial for improving depression.

In addition, FMT in SPF and GF mice confirmed the role of gut microbes in the development and treatment of depression. By performing FMT in healthy SPF and GF mice, the effect of the gut microbiota from depressed mice on the gut microbiome and behavioral regulation of healthy hosts was observed. For FMT in SPF mice, the microbiota of control mice was disturbed, rather than replaced by the microbiota from CUMS mice. At the phylum level, inconsistent with the stress-induced changes in the gut microbiota, no significant increase was observed in the abundance of Proteobacteria after CUMS-FMT. However, at the genus level, a decrease in *Lactobacillus* abundance and an increased abundance of *Bacteroides* was observed, which is consistent with changes in CUMS-induced mice. The reason for this phenomenon may be that during the FMT process, the host’s original gut microbiota has a certain resistance to the CUMS microbiota; therefore, the changes at the phylum level are not completely consistent with the changes in the microbiota of CUMS mice. Therefore, we performed CUMS-FMT in GF mice. Consistent with our expectations, the gut microbial signature (including the increased abundance of Proteobacteria at the phylum level and *Bacteroides* at the genus level) of CUMS mice was replicated in GF mice owing to the absence of resident microbiota in the gut. Both SPF and GF mice exhibited obvious depression-like behavior after CUMS-FMT treatment. This further confirms that perturbations in the microbiota, even at the genus level, are sufficient to affect the overall gut microbiome imbalances and induce the emergence of depressive-like behaviors. In this study, FMT was exclusively performed from CUMS mice to GF mice; FMT from control SPF mice to GF mice was not conducted. Recent high-quality references consistently demonstrate that FMT from unstressed animals does not induce depressive behavior in GF mice [47–49]. Earlier studies have indicated that GF mice did not display depression-like behaviors even after receiving fecal bacteria from control mice or healthy individuals. This suggests that fecal bacteria transplanted from unstressed mice or individuals did not influence the behavior of GF mice [47.48]. Next, the modulating effect of XYS-FMT on gut microbes was observed in CUMS mice. At the phylum level, XYS-FMT reduced the abundance of Proteobacteria and increased the abundance of Bacteroidetes, which is consistent with the regulation of gut microbiota by XYS administration. At the genus level, an increase in the abundance of *Lactobacillus* was observed. More importantly, changes in Prevotellaceae at the family level and *Prevotella* at the genus level were observed. Members of Prevotellaceae, known as gut symbionts, can produce SCFAs through dietary fiber fermentation and intestinal mucins [50]. Studies have shown that the reduction of Prevotellaceae results in increased exposure to the bacterial endotoxin system and increased intestinal permeability, inducing inflammation [51]. In this study, the LDA analysis showed that XYS-FMT increased the abundance of Prevotellaceae at the family level and *Prevotella* at the genus level, suggesting that XYS-FMT improves gut microbial structure and diversity in CUMS. An increase in Prevotellaceae at the family level was also observed in the CUMS-FMT group, but this increase did not lead to the disappearance of depression-like behaviors. The reason is that the changes in Prevotellaceae were not dominant in the microbial changes of the CUMS-FMT group, especially with the decrease of *Lactobacillus* and increase of *Bacteroides*; the complex and unbalanced micro-ecosystem led to the appearance of depression-like behaviors. In addition, the heterogeneity of Prevotellaceae at the species level may lead to diverse functions. For example, in cynomolgus monkeys with depression-like behaviors induced by CUMS, 11 increased amplicon sequence variants (ASVs) and 7 decreased ASVs belonged to Prevotellaceae, suggesting the complexity of Prevotellaceae species-level changes in depression-like behaviors [52]. However, in XYS-FMT, changes in Prevotellaceae were dominant in the microflora changes, which indicated that it may have a more significant inhibitory effect on inflammation. In conclusion, alterations in the gut microbial composition and diversity are strongly associated with the progression and treatment of depression-like behaviors. The effect of exogenous microbial supplementation on endogenous gut microbiota is mainly achieved by regulating the overall microbial environment and reshaping the abundance of several dominant bacteria rather than only affecting a single genus.

Crosstalk between gut microbes and host behavior is also reflected in the disruption of gut barrier function and regulation of microbial metabolites [53]. Intestinal homeostasis is maintained by crosstalk between the microbiota, intestinal barrier, and immune system [54]. An imbalance in the microbiome often leads to the destruction of the intestinal barrier and immune system. Under normal physiological conditions, intestinal epithelial cells, microbiomes, and immune cells support a steady state in the intestinal system. Intestinal epithelial cells receive signals from the microbiome, such as microbial metabolites or microbes, to preserve the mucosal barrier [55]. The microbiome also regulates host immunity through its metabolites and endotoxins. Furthermore, immune cells can directly or indirectly affect the microbiome by releasing cytokines or chemokines [56]. However, an imbalance in the microbiome leads to the destruction of the intestinal barrier and the over-activation of intestinal immunity. Studies have shown that a shift in microbiome structure leads to an increase in intestinal tight junction proteins and intestinal permeability. These events induce inflammation in the colon and produce proinflammatory cytokines such as IL-6, IL-1β, and TNF-α [25]. In this study, we observed that CUMS or CUMS-FMT could lead to the disruption of colonic barrier function, production of colonic inflammation, and elevation of serum proinflammatory cytokines IL-6, IL-1β, and TNF-α, whereas XYS-FMT can restore intestinal barrier function and inhibit colon inflammation and inflammatory factors in sera. Metabolism is closely related to homeostasis of the intestinal barrier function. Metabolism is a key pathway through which the intestinal flora affects depression via the brain-gut axis, especially through direct changes in the levels of key metabolites and indirect alterations in circulating serum metabolites, thereby further affecting changes in the CNS that regulate depressive behaviors [57]. Consistent with the changes in the microbiota, disturbances in microbial metabolites were observed under two different conditions (stress and microbial perturbation). Vitamin B6 metabolism and lysine biosynthesis pathways showed significantly different enrichment in the current study. Vitamin B6 deficiency is associated with depression and adverse neurological function [58]. In addition, research has shown that premedication with vitamin B6 could prevent dexamethasone-induced depression and demonstrate antidepressant effects [59]. Lysine is an important factor in gut microbiota-mediated depression. A previous study suggested that altered lysine acetylation may play a key role in the development of depression and may be a mechanism by which the gut microbiota modulates brain function and behavioral phenotypes [60]. In the current study, we found that the progression of depression-like behavior was accompanied by disturbances in vitamin B6 metabolism and lysine biosynthesis, and correlation analysis also revealed the connection among microbial metabolites, behavior, and intestinal flora. Using changes in the abundance of Proteobacteria as a feature of microbial disturbances, UPLC-MS-based analysis detected changes in substrates for the production of multiple bacterial endotoxins (LPS). LPS, a substance (glycolipid) composed of lipids and polysaccharides, is a constituent of the outer cell wall of gram-negative bacteria [61]. High levels of LPS in the peripheral blood and CNS are thought to be associated with depression [62]. It has been reported that endotoxin levels increase three-fold in the blood and two- or three-fold in the brains of patients with depression. D-( +)-Mannose, N-acetylmannosamine, D-ribose 5-phosphate, and glucose-6-phosphate are important substrates for LPS synthesis. In the present study, an increase in the consumption of these substrates was observed, suggesting an increase in the production of LPS. This is consistent with a previous report of increased LPS-biosynthesis gene expression in patients with anxiety and depression [62]. In addition, with the disruption of the intestinal barrier function, a high level of LPS expression was observed in peripheral blood, which is consistent with the increased abundance of LPS-producing bacteria in the gut microbiota. Correlation analysis also confirmed that the gut microbes were highly correlated with LPS production.

Overexposure to LPS induces expression of the complement system and activation of microglia [63]. Complement, a serum protein, has enzymatic activity and mediates immune and inflammatory responses. Complement C3 is a key protein in the complement system [64]. Elevated levels of C3 have been observed in the brains of patients with depression and animal depression models and are thought to be positively correlated with the severity of depressive symptoms [65]. Researchers have found that chronic stress can lead to increased expression of C3 in the PFC of mice, and C3-KO mice do not develop depression-like behaviors, suggesting that a high level of C3 is an important condition for the onset of depression [15]. Unfortunately, the source of complement C3 (either directly from the CNS or via inflammatory migration from the periphery to the brain) remains unclear. LPS is thought to be a key activator of complement C3, mediating the levels of complement C3 in peripheral sera [66]. After observing gut microbiota-regulated LPS elevation, C3 expression in the sera and PFC of mice were examined. Consistent with stress-induced complement changes, high expression of C3 in the peripheral and prefrontal cortices after fecal transplantation suggests that gut microbial disturbances may be a trigger and aggravating factor for C3 activation. In addition, the activation of microglia specifically induced by LPS in the CNS was observed. As innate immune effector cells in the CNS, microglia play an important role in maintaining neuronal development and normal neural function [67]. Among them, synapse pruning is the main method for microglia to eliminate synapses and is an important mechanism for the selective pruning of neurons to maintain normal neuronal systems [68]. Crosstalk with the complement system, which mediates microglial synapse pruning, is critical for the regulation of synaptic morphology by microglia [69]. Recent research has shown that overexpression of complement C3 affects phagocytosis and synaptic pruning of microglia, leading to depression and abnormal cognitive behavior [70]. CR3 is a complement C3 receptor expressed by microglia, and its binding to complement C3 guides microglia to phagocytose-labeled neuronal synapses [71]. In this study, either stress or transplantation of dysregulated gut microbiota could induce activation of the C3/CR3 pathway in the PFC and reduce the protein expression levels of SYN and PSD95. Antidepressants or antidepressant-FMT can inhibit the activation of the C3/CR3 pathway and prevent the reduction of SYN and PSD95 expression. SYN and PSD95 are important indicators reflecting the synaptic function of neurons, and low SYN and PSD95 expression is considered an important pathological feature of depression [72].

**Fig. 10:**
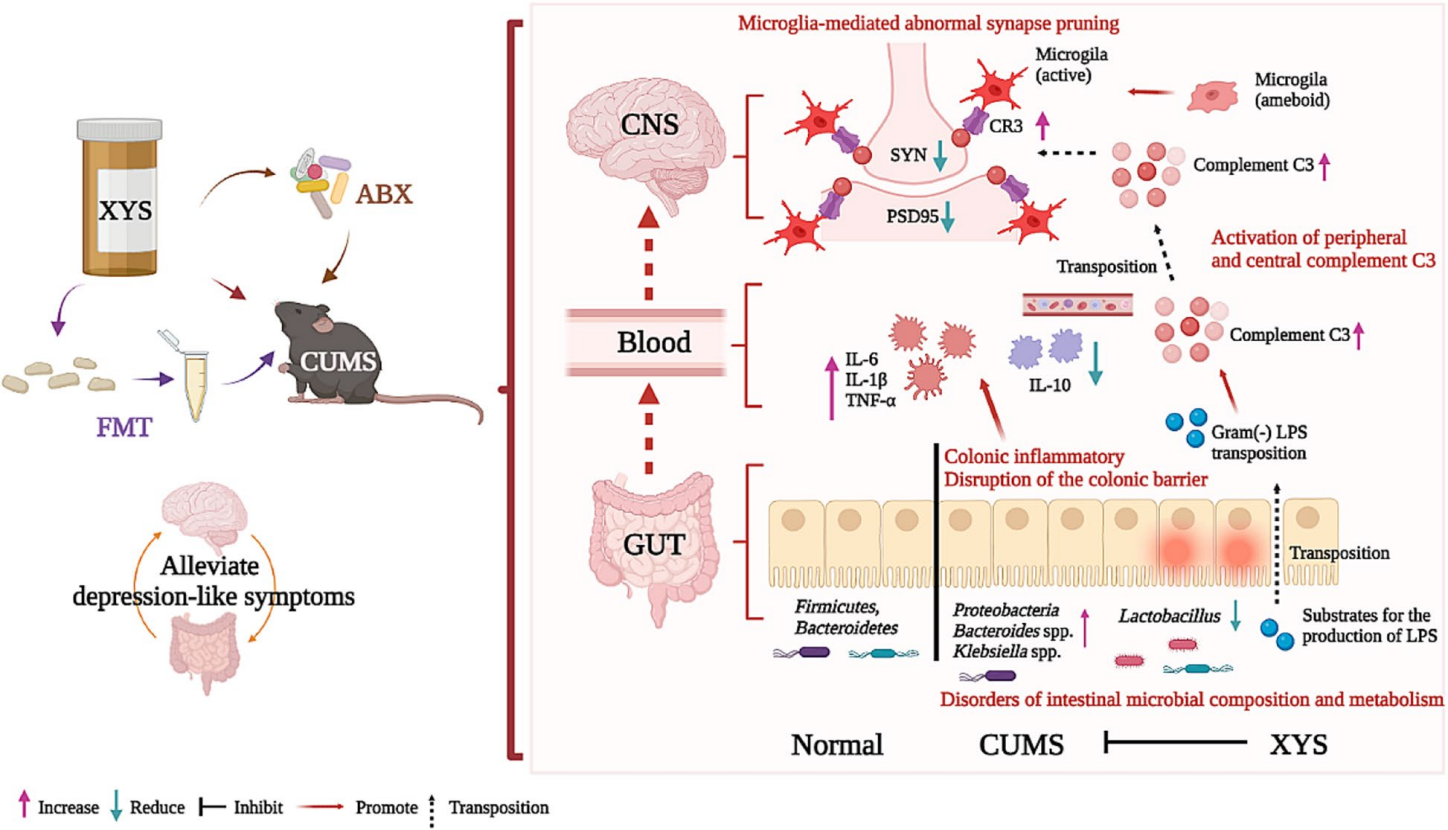
Inhibition of abnormal synaptic pruning is the key to targeting microbes to treat depression. Gut microbiota contributes to regulating the gut-brain axis and maintaining health, while its alteration (increase of *Proteobacteria* and decreased of microbial diversity) due to CUMS is related to depression and its adverse consequences on cognition. XYS supplementation is thought to decrease *Proteobacteria* and regulated the composition and metabolism of gut microbiota (1), thereby, contribute to gut barrier function and immune homeostasis (2); this attenuates the translocation of components of Gram-negative bacteria (3), which decreases the peripheral inflammatory tone and inhibits activation of C3/CR3 (4) and microglia-mediated abnormal synaptic pruning in the CNS (5). Therefore, the supplement of XYS beneficially impacts on depression, via restoration of gut microbiota and its regulatory role in the gut-brain axis, suggesting that inhibition of abnormal synaptic pruning is the key to targeting microbes to treat depression

Collectively, our results suggest that gut dysbiosis induces depression-like behaviors through abnormal synapse pruning in microglia, mediated by complement C3. Furthermore, antidepressant-mediated alterations in the microbiome, especially inhibition of Proteobacteria, play a key role in the alleviation of depression. By regulating complement C3 and microglia involved in the gut-brain crosstalk pathway in the pathogenesis of depression, the inhibition of abnormal synaptic pruning becomes the key to targeting microbes to treat depression. These results are summarized in Fig. 10.

## Conclusion

In summary, our results provide evidence that gut microbiota restoration can regulate C3/CR3-mediated synaptic pruning and exhibit antidepressant activity through brain-gut interactions. Antidepressants attenuate CUMS-induced depression, and their different impacts on the composition and metabolites of gut microbiota imply a central role of gut microbiota in mediating beneficial effects. Improvements in depression-like behaviors are believed to be mediated mainly by modulation of the gut microbiota, suppression of the abundance of LPS-producing bacteria, and subsequent inhibition of LPS production. These changes in the gut microbiota subsequently led to the inhibition of complement C3 activation, which in turn ameliorates the program of complement disturbance and central complement-mediated abnormal synaptic pruning in microglia, resulting in the attenuation of depression symptoms. These findings provide novel insights into gut microbiota-mediated alleviation of depression and will facilitate the development of therapeutic and preventive strategies for depression and other mental disorders.

### Supplementary Information

**Additional file 2. **

**Additional file 3. **

**Additional file 4. **

**Additional file 5: Supplement Fig. 1. **Pretreatment with antibiotics attenuated the antidepressant effect of XYS. A: Body weight in week 0. B: SPT in week 0. C: OFT in week 0. D: Body weight in week 8. E: SPT in week 8. F: OFT in week 8. G: Movement trajectories of mice in OFT in week 0 and week 8. H: The study design of antibiotics interfering with the antidepressant effect of XYS. I: SPT. J: OFT. Data represent the mean ± SEM (n=10 per group). ^**^P < 0.01, ^***^P < 0.001 versus the Control group; ^##^*P*< 0.01, ^###^*P* < 0.001 versus the CUMS group.

**Additional file 6: Supplement Fig. 2.** Depression-like behaviors are accompanied by dysbiosis in gut homeostasis, and antidepressants restore gut homeostasis. Alpha diversity of A: chao1 index; B: observed index; C: simpson index. D: Analysis of differences in the microbial taxa at phylum level shown by LEfSe (linear discriminant analysis coupled with effect size measurements). E: Microbiota community of rats at the genus level, the relative abundance rates of each bacterial group. The relative abundance variation of each genus in normal group, CUMS group, XYS group and FLX group is indicated by a gradient of color from green (low) to red (high). Fecal bacteria distributed by Partial Least Squares Discriminant Analysis (PLS-DA) based on weighted UniFrac distance, between F: CUMS versus CUMS+XYS. G: Control versus CUMS. H: CUMS versus CUMS+FLX. KEGG enrichment results of differential metabolites in normal group, CUMS group, XYS group and FLX group of I: positive mode; J: negative mode. K: LPS-generated substrates. L: D-(+)-Mannose. M: N-Acetylmannosamine. N: Glucose-6-phosphate. O: D-ribose5-phosphate. Data represent the mean ± SEM (n=6 per group). ^***^P < 0.001 versus the Control group; ^###^*P*< 0.001 versus the CUMS group.

**Additional file 7: Supplement Fig. 3.** Abnormal activation of microglia is important pathological manifestations of depression. A: Activation of CD68 and iba-1-labeled microglia (Control, CUMS, CUMS+XYS and CUMS+FLX). B: Activation of CD68 and iba-1-labeled microglia (Control, Control+FMT(CUMS), CUMS and CUMS+FMT(XYS)). C: Activation of CD68 and iba-1-labeled microglia (GF, GF+FMT(CUMS)). Data represent the mean ± SEM (n=3 per group). ^***^P< 0.001 versus the Control group; ^###^*P* < 0.001 versus the CUMS group.

**Additional file 8: Supplement Fig. 4.** Transplantation of dysregulated gut microbiota directly induces development of depression-like behaviors. A: Study design. B: SPT in week 0. C: SPT in week 8. D: OFT in week 0. E: OFT in week 8. F: Movement trajectories of mice in OFT. Data represent the mean ± SEM (n=10 per group). ^*^P < 0.05, ^**^P < 0.01, ^***^P< 0.001 versus the Control group; ^#^*P* < 0.05, ^##^*P*< 0.01, ^###^*P* < 0.001 versus the CUMS group.

**Additional file 9: Supplement Fig. 5.** CUMS-FMT mediated disturbance of the gut microbiota in mice; XYS-FMT regulated the composition of gut microbiota. Alpha diversity shown by A: simpson index. B: observed index. C: chao1 index. D: Gut microbiota change at genus level (Control versus CUMS). E: Gut microbiota change at genus level (Control versus Control+FMT(CUMS)). F: Gut microbiota change at genus level (CUMS versus CUMS+FMT(XYS)).

## Data Availability

The sequencing data of the 16S rRNA gene in this study are available in the Sequence Read Archive (SRA) under project number PRJNA913473.

## Abbreviations

ABX: Antibiotics
C3: Component 3
CUMS: Chronic unpredictable mild stress
CR3: Complement receptors 3
CNS: Central nervous system
EPM: Elevated plus-maze test
ELISA: Enzyme-linked immunosorbent assay
FMT: Fecal microbiota transplantation
FLX: Fluoxetine
GF: Germ-free
HE: Hematoxylin and eosin
IF: Immunofluorescence
IL: Interleukin
KEGG: Kyoto Encyclopedia of Genes and Genomes
LEfSe: Linear discriminant analysis effect size
LPS: Lipopolysaccharide
NORT: Novel object recognition test
OFT: Open field test
OPLS-DA: Orthogonal partial least squares-discriminant analysis
PBS: Phosphate-buffered saline
PLS-DA: Partial least squares-discriminant analysis
PCoA: Principal coordinate analysis
PFC: Prefrontal cortex
PSD95: Postsynaptic density 95
SPF: Specific pathogen-free
SPT: Sucrose preference test
SYN: Synapsin
TST: Tail suspension test
TNF: Tumor necrosis factor
UhPLC-QTOF-MS: Ultra-high performance liquid chromatography-quadrupole time-of-flight mass spectrometry
WHO: World Health Organization
WB: Western blotting
XYS: Xiaoyaosan

## References

[1] Wolf J, Padberg F, Nenov-Matt T, Amann BL, Barton BB, Tang J (2022). Suicidal behaviors are associated with loneliness and decrease during inpatient CBASP treatment for persistent depressive disorder. J Psychiatr Res.

[2] Sender R, Fuchs S, Milo R (2016). Are we really vastly outnumbered? Revisiting the ratio of bacterial to host cells in humans. Cell.

[3] Dinan TG, Cryan JF (2017). Gut instincts: microbiota as a key regulator of brain development, ageing and neurodegeneration. J Physiol.

[4] Wu X, Xia Y, He F, Zhu C (2021). Intestinal mycobiota in health and diseases: from a disrupted equilibrium to clinical opportunities. Microbiome.

[5] Tian T, Mao Q, Xie J, Wang Y, Shao WH, Zhong Q (2022). Multi-omics data reveals the disturbance of glycerophospholipid metabolism caused by disordered gut microbiota in depressed mice. J Adv Res.

[6] Simkin DR (2019). Microbiome and mental health, specifically as it relates to adolescents. Curr Psychiatry Rep.

[7] Lahtinen P, Jalanka J, Hartikainen A, Mattila E, Hillilä M, Punkkinen J (2020). Randomised clinical trial: faecal microbiota transplantation versus autologous placebo administered via colonoscopy in irritable bowel syndrome. Aliment Pharmacol Ther.

[8] Zhang YQ, Lin WP, Huang LP, Zhao B, Zhang CC, Yin DM (2021). Dopamine D2 receptor regulates cortical synaptic pruning in rodents. Nat Commun.

[9] Duman RS, Aghajanian GK (2012). Synaptic dysfunction in depression: potential therapeutic targets. Science.

[10] Hong S, Beja-Glasser VF, Nfonoyim BM, Frouin A, Li S, Ramakrishnan S (2016). Complement and microglia mediate early synapse loss in Alzheimer mouse models. Science.

[11] Zhang MM, Guo MX, Zhang QP, Chen XQ, Li NZ, Liu Q (2022). IL-1R/C3aR signaling regulates synaptic pruning in the prefrontal cortex of depression. Cell Biosci.

[12] Boulanger LM (2009). Immune proteins in brain development and synaptic plasticity. Neuron.

[13] Geisbrecht BV, Lambris JD, Gros P (2022). Complement component C3: a structural perspective and potential therapeutic implications. Semin Immunol.

[14] Cornell J, Salinas S, Huang HY, Zhou M (2022). Microglia regulation of synaptic plasticity and learning and memory. Neural Regen Res.

[15] Crider A, Feng T, Pandya CD, Davis T, Nair A, Ahmed AO (2018). Complement component 3a receptor deficiency attenuates chronic stress-induced monocyte infiltration and depressive-like behavior. Brain Behav Immun.

[16] Berkowitz S, Chapman J, Dori A, Gofrit SG (2021). Complement and coagulation system crosstalk in synaptic and neural conduction in the central and peripheral nervous systems. Biomedicines.

[17] Bi C, Guo S, Hu S, Chen J, Ye M, Liu Z (2022). The microbiota–gut–brain axis and its modulation in the therapy of depression: comparison of efficacy of conventional drugs and traditional Chinese medicine approaches. Pharmacol Res.

[18] Hao W, Gan H, Wang L, Huang J, Chen J (2023). Polyphenols in edible herbal medicine: targeting gut-brain interactions in depression-associated neuroinflammation. Crit Rev Food Sci Nutr..

[19] Hao W, Wu J, Yuan N, Gong L, Huang J, Ma Q (2021). Xiaoyaosan improves antibiotic-induced depressive-like and anxiety-like behavior in mice through modulating the gut microbiota and regulating the NLRP3 inflammasome in the colon. Front Pharmacol.

[20] Chen J, Lei C, Li X, Wu Q, Liu C, Ma Q (2022). Research progress on classical traditional Chinese medicine formula xiaoyaosan in the treatment of depression. Front Pharmacol.

[21] Chan K, Lee H. The progress of Chinese medicine in the United Kingdom[M]//The Way Forward for Chinese Medicine. Routledge; 2001. p. 317-344.

[22] Wang Q, Gao S, Zhang W, Zhao Y, He Y, Sun W (2022). Traditional use and safety evaluation of combination Traditional Chinese Medicine in European registration: with XiaoYao Tablets as an example. Pharmazie.

[23] Commission GBM, GM. Council, British pharmacopoeia. General Medical Council. 1864.

[24] Zhu HZ, Liang YD, Ma QY, Hao WZ, Li XJ, Wu MS (2019). Xiaoyaosan improves depressive-like behavior in rats with chronic immobilization stress through modulation of the gut microbiota. Biomed Pharmacother.

[25] Hao WZ, Ma QY, Tao G, Huang JQ, Chen JX (2021). Oral coniferyl ferulate attenuated depression symptoms in mice via reshaping gut microbiota and microbial metabolism. Food Funct.

[26] O'Leary OF, Felice D, Galimberti S, Savignac HM, Bravo JA, Crowley T, El Yacoubi M, Vaugeois JM, Gassmann M, Bettler B, Dinan TG, Cryan JF (2014). GABAB(1) receptor subunit isoforms differentially regulate stress resilience. Proc Natl Acad Sci U S A.

[27] van de Wouw M, Walsh AM, Crispie F, van Leuven L, Lyte JM (2020). Distinct actions of the fermented beverage kefir on host behaviour, immunity and microbiome gut-brain modules in the mouse. Microbiome.

[28] Wu Z, Huang S, Li T, Li N, Han D (2021). Gut microbiota from green tea polyphenol-dosed mice improves intestinal epithelial homeostasis and ameliorates experimental colitis. Microbiome.

[29] Shi H, Yu Y, Lin D, Zheng P, Zhang P (2020). β-glucan attenuates cognitive impairment via the gut-brain axis in diet-induced obese mice. Microbiome.

[30] Gershon MD, Margolis KG (2021). The gut, its microbiome, and the brain: connections and communications. J Clin Invest.

[31] Palepu MSK, Dandekar MP (2022). Remodeling of microbiota gut-brain axis using psychobiotics in depression. Eur J Pharmacol.

[32] Schaub AC, Schneider E, Vazquez-Castellanos JF, Schweinfurth N, Kettelhack C, Doll JPK (2022). Clinical, gut microbial and neural effects of a probiotic add-on therapy in depressed patients: a randomized controlled trial. Transl Psychiatry.

[33] Guida F, Turco F, Iannotta M, De Gregorio D, Palumbo I, Sarnelli G (2018). Antibiotic-induced microbiota perturbation causes gut endocannabinoidome changes, hippocampal neuroglial reorganization and depression in mice. Brain Behav Immun.

[34] Ilgin S, Can OD, Atli O, Ucel UI, Sener E, Guven I (2015). Ciprofloxacin-induced neurotoxicity: evaluation of possible underlying mechanisms. Toxicol Mech Methods.

[35] Łoniewski I, Misera A, Skonieczna-Żydecka K, Kaczmarczyk M, Kaźmierczak-Siedlecka K, Misiak B (2021). Major depressive disorder and gut microbiota–association not causation. A scoping review. Prog Neuropsychopharmacol Biol Psychiatry..

[36] Kosuge A, Kunisawa K, Arai S, Sugawara Y, Shinohara K, Iida T (2021). Heat-sterilized Bifidobacterium breve prevents depression-like behavior and interleukin-1β expression in mice exposed to chronic social defeat stress. Brain Behav Immun.

[37] Shin NR, Whon TW, Bae JW (2015). Proteobacteria: microbial signature of dysbiosis in gut microbiota. Trends Biotechnol.

[38] Morgan XC, Tickle TL, Sokol H, Gevers D, Devaney KL, Ward DV (2012). Dysfunction of the intestinal microbiome in inflammatory bowel disease and treatment. Genome Biol.

[39] Simpson CA, Mu A, Haslam N, Schwartz OS, Simmons JG (2020). Feeling down? A systematic review of the gut microbiota in anxiety/depression and irritable bowel syndrome. J Affect Disord.

[40] Zhang Y, Liang H, Wang Y, Cheng R, Pu F, Yang Y (2022). Heat-inactivated Lacticaseibacillus paracasei N1115 alleviates the damage due to brain function caused by long-term antibiotic cocktail exposure in mice. BMC Neurosci.

[41] Jang HM, Kim JK, Joo MK, Shin YJ, Lee KE, Lee CK (2022). Enterococcus faecium and Pediococcus acidilactici deteriorate Enterobacteriaceae-induced depression and colitis in mice. Sci Rep.

[42] Tamana SK, Tun HM, Konya T, Chari RS, Field CJ, Guttman DS (2021). Bacteroides-dominant gut microbiome of late infancy is associated with enhanced neurodevelopment. Gut Microbes.

[43] Zhang Y, Fan Q, Hou Y, Zhang X, Yin Z, Cai X (2022). Bacteroides species differentially modulate depression-like behavior via gut-brain metabolic signaling. Brain Behav Immun.

[44] Yang J, Zheng P, Li Y, Wu J, Tan X, Zhou J (2020). Landscapes of bacterial and metabolic signatures and their interaction in major depressive disorders. Sci Adv.

[45] Romijn AR, Rucklidge JJ, Kuijer RG, Frampton C (2017). A double-blind, randomized, placebo-controlled trial of Lactobacillus helveticus and Bifidobacterium longum for the symptoms of depression. Aust N Z J Psychiatry.

[46] Zheng P, Zeng B, Zhou C, Liu M, Fang Z, Xu X (2016). Gut microbiome remodeling induces depressive-like behaviors through a pathway mediated by the host’s metabolism. Mol Psychiatry.

[47] De Palma G, Blennerhassett P, Lu J, Deng Y, Park AJ (2015). Microbiota and host determinants of behavioural phenotype in maternally separated mice. Nat Commun.

[48] Zheng P, Zeng B, Zhou C, Liu M, Fang Z (2016). Gut microbiome remodeling induces depressive-like behaviors through a pathway mediated by the host's metabolism. Mol Psychiatry.

[49] Luo YY, Zeng BH, Zeng L, Du XY, Li B (2018). Gut microbiota regulates mouse behaviors through glucocorticoid receptor pathway genes in the hippocampus. Transl Psychiatry.

[50] Sorboni SG, Moghaddam HS, Jafarzadeh-Esfehani R, Soleimanpour S (2022). A comprehensive review on the role of the gut microbiome in human neurological disorders. Clin Microbiol Rev.

[51] Rosario D, Bidkhori G, Lee S, Bedarf J, Hildebrand F, Le Chatelier E (2021). Systematic analysis of gut microbiome reveals the role of bacterial folate and homocysteine metabolism in Parkinson’s disease. Cell Rep.

[52] Teng T, Clarke G, Maes M, Jiang Y, Wang J, Li X (2022). Biogeography of the large intestinal mucosal and luminal microbiome in cynomolgus macaques with depressive-like behavior. Mol Psychiatry.

[53] Krautkramer KA, Fan J, Bäckhed F (2021). Gut microbial metabolites as multi-kingdom intermediates. Nat Rev Microbiol.

[54] Kayama H, Okumura R, Takeda K (2020). Interaction between the microbiota, epithelia, and immune cells in the intestine. Annu Rev Immunol.

[55] Nadjsombati MS, McGinty JW, Lyons-Cohen MR, Jaffe JB, DiPeso L, Schneider C (2018). Detection of succinate by intestinal tuft cells triggers a type 2 innate immune circuit. Immunity.

[56] Levy M, Kolodziejczyk AA, Thaiss CA, Elinav E (2017). Dysbiosis and the immune system. Nat Rev Immunol.

[57] Hao WZ, Li XJ, Zhang PW, Chen JX (2020). A review of antibiotics, depression, and the gut microbiome. Psychiatry Res.

[58] Berkins S, Schiöth HB, Rukh G (2021). Depression and vegetarians: association between dietary vitamin B6, B12 and folate intake and global and subcortical brain volumes. Nutrients.

[59] Mesripour A, Alhimma F, Hajhashemi V (2019). The effect of vitamin B6 on dexamethasone-induced depression in mice model of despair. Nutr Neurosci.

[60] Yu Y, Wang H, Rao X, Liu L, Zheng P, Li W (2021). Proteomic profiling of lysine acetylation indicates mitochondrial dysfunction in the hippocampus of gut microbiota-absent mice. Front Mol Neurosci.

[61] Raetz CR, Whitfield C (2002). Lipopolysaccharide endotoxins. Annu Rev Biochem.

[62] Stevens BR, Goel R, Seungbum K, Richards EM, Holbert RC, Pepine CJ (2018). Increased human intestinal barrier permeability plasma biomarkers zonulin and FABP2 correlated with plasma LPS and altered gut microbiome in anxiety or depression. Gut.

[63] Bodea LG, Wang Y, Linnartz-Gerlach B, Kopatz J, Sinkkonen L, Musgrove R (2014). Neurodegeneration by activation of the microglial complement–phagosome pathway. J Neurosci.

[64] Götze O, Müller-Eberhard HJ (1971). The C3-activator system: an alternate pathway of complement activation. J Exp Med.

[65] Tao H, Chen X, Zhou H, Fu J, Yu Q, Liu Y (2020). Changes of serum melatonin, Interleukin-6, Homocysteine, and Complement C3 and C4 levels in patients with depression. Front Psychol.

[66] Morrison DC, Kline LF (1977). Activation of the classical and properdin pathways of complement by bacterial lipopolysaccharides (LPS). J Immunol.

[67] Hong S, Dissing-Olesen L, Stevens B (2016). New insights on the role of microglia in synaptic pruning in health and disease. Curr Opin Neurobiol.

[68] Paolicelli RC, Bolasco G, Pagani F, Maggi L, Scianni M, Panzanelli P (2011). Synaptic pruning by microglia is necessary for normal brain development. Science.

[69] Stephan AH, Barres BA, Stevens B (2012). The complement system: an unexpected role in synaptic pruning during development and disease. Annu Rev Neurosci.

[70] Germann M, Brederoo SG, Sommer IEC (2021). Abnormal synaptic pruning during adolescence underlying the development of psychotic disorders. Curr Opin Psychiatry.

[71] Presumey J, Bialas AR, Carroll MC (2017). Complement system in neural synapse elimination in development and disease. Adv Immunol.

[72] Vatandoust SM, Meftahi GH (2022). The effect of sericin on the cognitive impairment, depression, and anxiety caused by learned helplessness in male mice. J Mol Neurosci.

