# Supplementary material for: Gut dysbiosis induces the development of depression-like behavior through abnormal synapse pruning in microglia-mediated by complement C3
Source: Microbiome. 2024 Feb 20;12:34. doi: 10.1186/s40168-024-01756-6 (PMC10877840; doi:10.1186/s40168-024-01756-6)
Supplement: Supplementary file 2 — Additional file 2. [file 40168_2024_1756_MOESM1_ESM.docx]

**Materials and methods**

**CUMS procedure**

The CUMS depression model was induced by daily exposure to alternating stressors for a continuous period of 8 weeks. (Supplement Table 1).

Supplement Table 1: Schedule of CUMS stimulation

| DAY | Food and water deprivation | Ice water swimming | Body restriction | Tail pinching | Day and night reversal | Heat stress | Foreign object stimulation |
| --- | --- | --- | --- | --- | --- | --- | --- |
| Monday | √ |  |  |  |  |  |  |
| Tuesday |  | √ |  |  |  |  |  |
| Wednesday |  |  | √ |  |  |  |  |
| Thursday |  |  |  | √ |  |  |  |
| Friday |  |  |  |  | √ |  |  |
| Saturday |  |  |  |  |  | √ |  |
| Sunday |  |  |  |  |  |  | √ |
