# Supplementary material for: Gut dysbiosis induces the development of depression-like behavior through abnormal synapse pruning in microglia-mediated by complement C3
Source: Microbiome. 2024 Feb 20;12:34. doi: 10.1186/s40168-024-01756-6 (PMC10877840; doi:10.1186/s40168-024-01756-6)
Supplement: Supplementary file 3 — Additional file 3. [file 40168_2024_1756_MOESM2_ESM.docx]

**The morphology of microglia**

The size of the microglial soma was increased in CUMS-induced mice with a more ramified phenotype. In addition, exposed to fecal bacteria from CUMS depressed mice also causes changes in microglia morphology (includingthe increased size of the microglial soma and ramified phenotype). Conversely, antidepressants and fecal microbiota transplantation from antidepressant-treated donors improved the abnormal morphology of microglia (Figure 1).


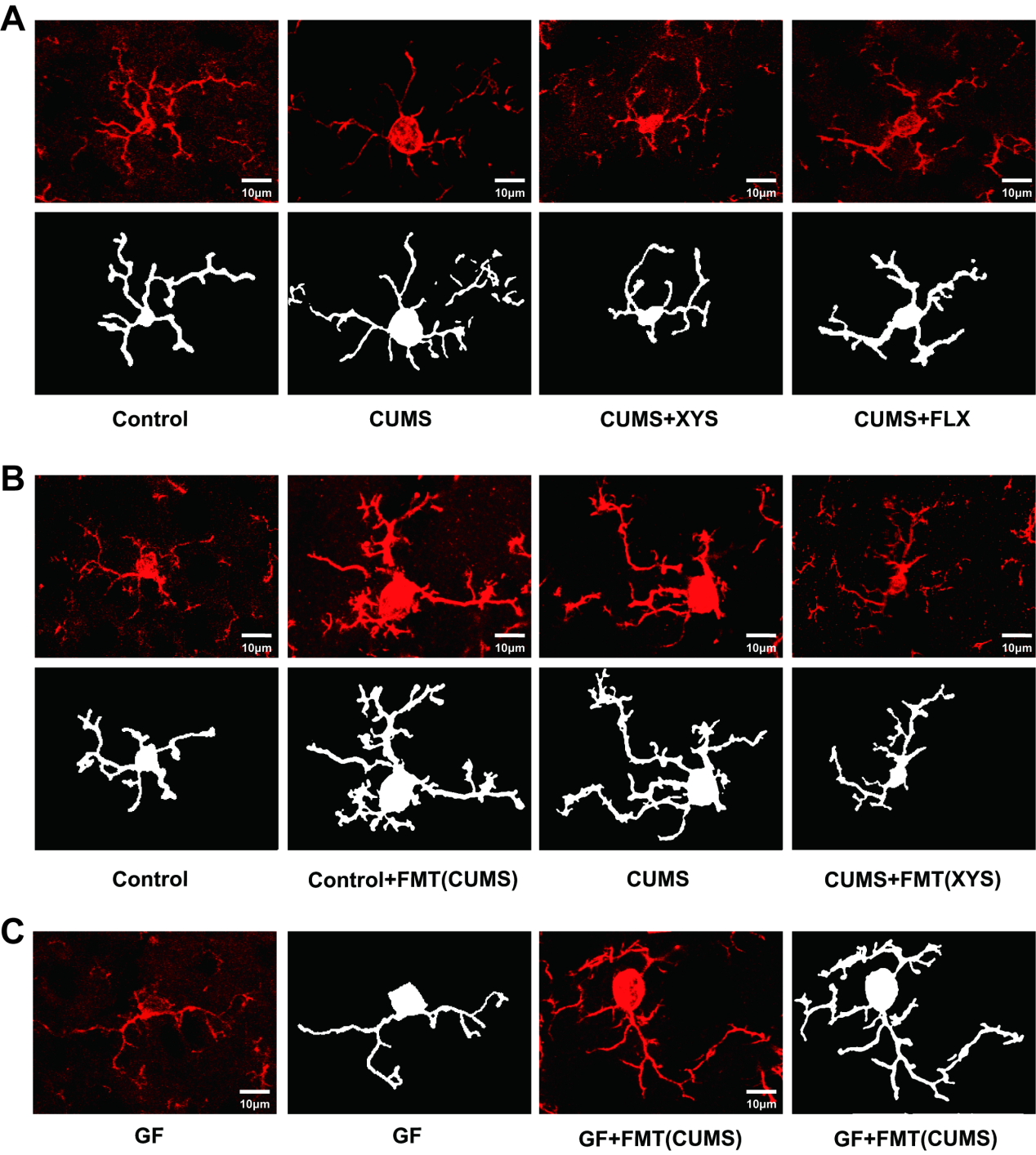


**Figure 1:** Microglia density (Iba1 staining, red) and morphology. The scale bar denotes 10 μm. A: Morphology of Iba-1-labeled microglia (Control, CUMS, CUMS+XYS and CUMS+FLX). B: Morphology of Iba-1-labeled microglia (Control, Control+FMT(CUMS), CUMS and CUMS+FMT(XYS)). C: Morphology of Iba-1-labeled microglia (GF, GF+FMT(CUMS)).
