# Supplementary material for: Gut dysbiosis induces the development of depression-like behavior through abnormal synapse pruning in microglia-mediated by complement C3
Source: Microbiome. 2024 Feb 20;12:34. doi: 10.1186/s40168-024-01756-6 (PMC10877840; doi:10.1186/s40168-024-01756-6)
Supplement: Supplementary file 4 — Additional file 4. [file 40168_2024_1756_MOESM3_ESM.docx]

**Methods**

**Animals and CUMS procedure**

Six- to eight-week-old SPF male C57BL/6 mice (SYXK (Yue) 2017-0174) weighing 20 ± 2 g were subjected to a seven-day adaptive feeding process. The experimental conditions included a room temperature of 21 ± 2 °C, relative humidity of 30–40%, and 12 h/12 h light/dark cycle. All mice in the study were individually housed. After one week of acclimation, mice were randomly divided into two groups: Control, Control+ABX. The treatments were as follows: (1) Control group: served as a negative control; (2) Control+ABX group: accepted an antibiotic cocktail consisting of ampicillin (1.5 g/mL; A9518; Sigma Aldrich), vancomycin (500 mg/L; V820413; Macklin), ciprofloxacin (200 mg/L; C9371; Solarbio), imipenem (250 mg/L; S27995), and metronidazole (1 g/L; B1976; Ape) in the mice's daily drinking water for one-week. The mice were immediately sacrificed after the last behavioral test. Through the TST, FST, and OFT tests, we observed that one week of ABX intervention did not induce abnormal behavior in mice (Figure 1). Our findings suggest that a one-week antibiotic treatment does not impact the behaviors of SPF mice.


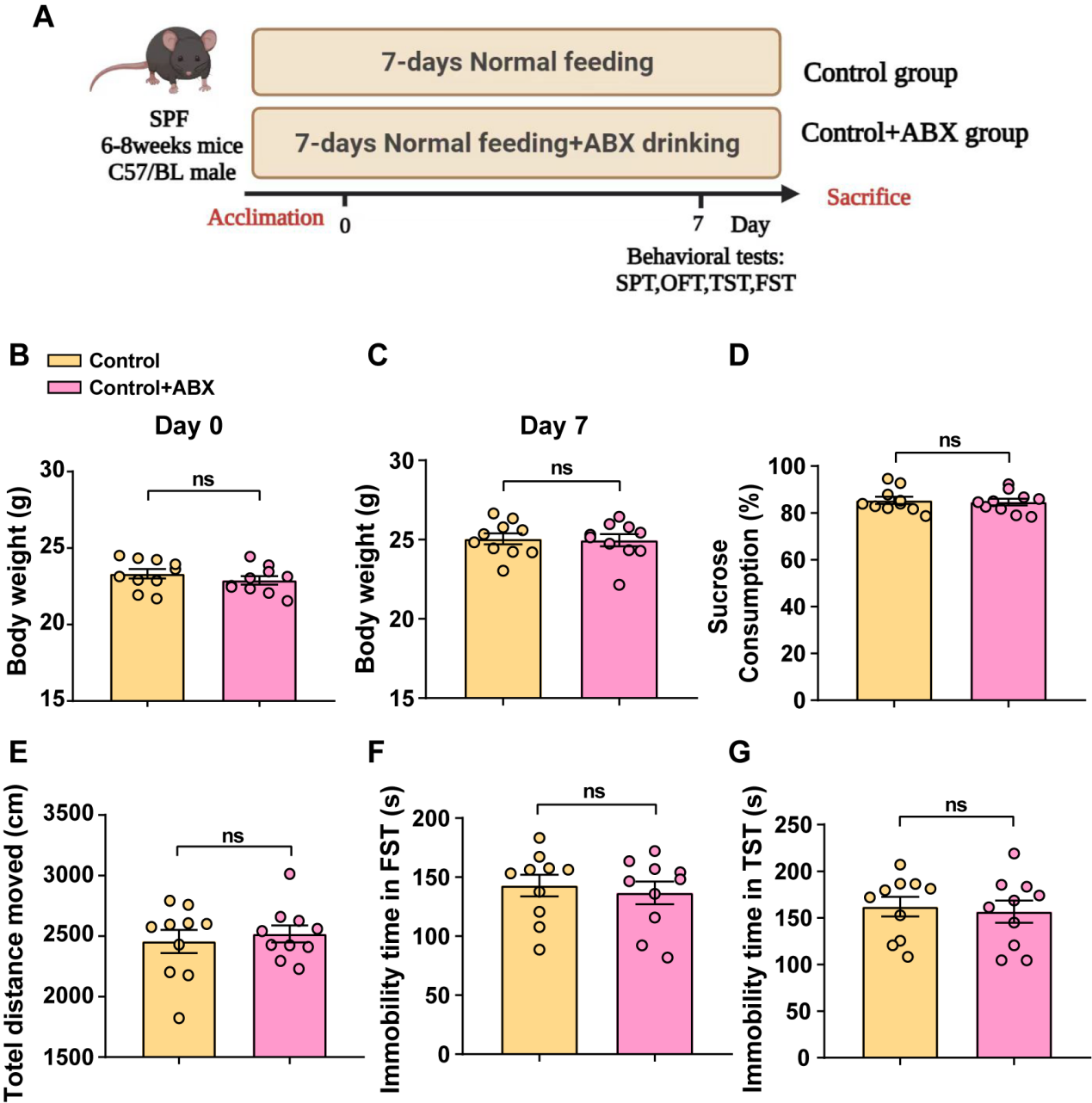


**Fig. 1** The 1-week ABX intervention did not significantly impact the behaviors of SPF mice. A: Study design. B: Body weight in Day 0. C: Body weight in Day 7. D: SPT in day 7. E: OFT. F: FST. G: TST. Data represent the mean ± SEM (n=10 per group). ^**^*P* < 0.01, ^***^*P* < 0.001 versus the Control group.
