# Supplementary figures and images for: Gut dysbiosis induces the development of depression-like behavior through abnormal synapse pruning in microglia-mediated by complement C3
Source: Microbiome. 2024 Feb 20;12:34. doi: 10.1186/s40168-024-01756-6 (PMC10877840; doi:10.1186/s40168-024-01756-6)

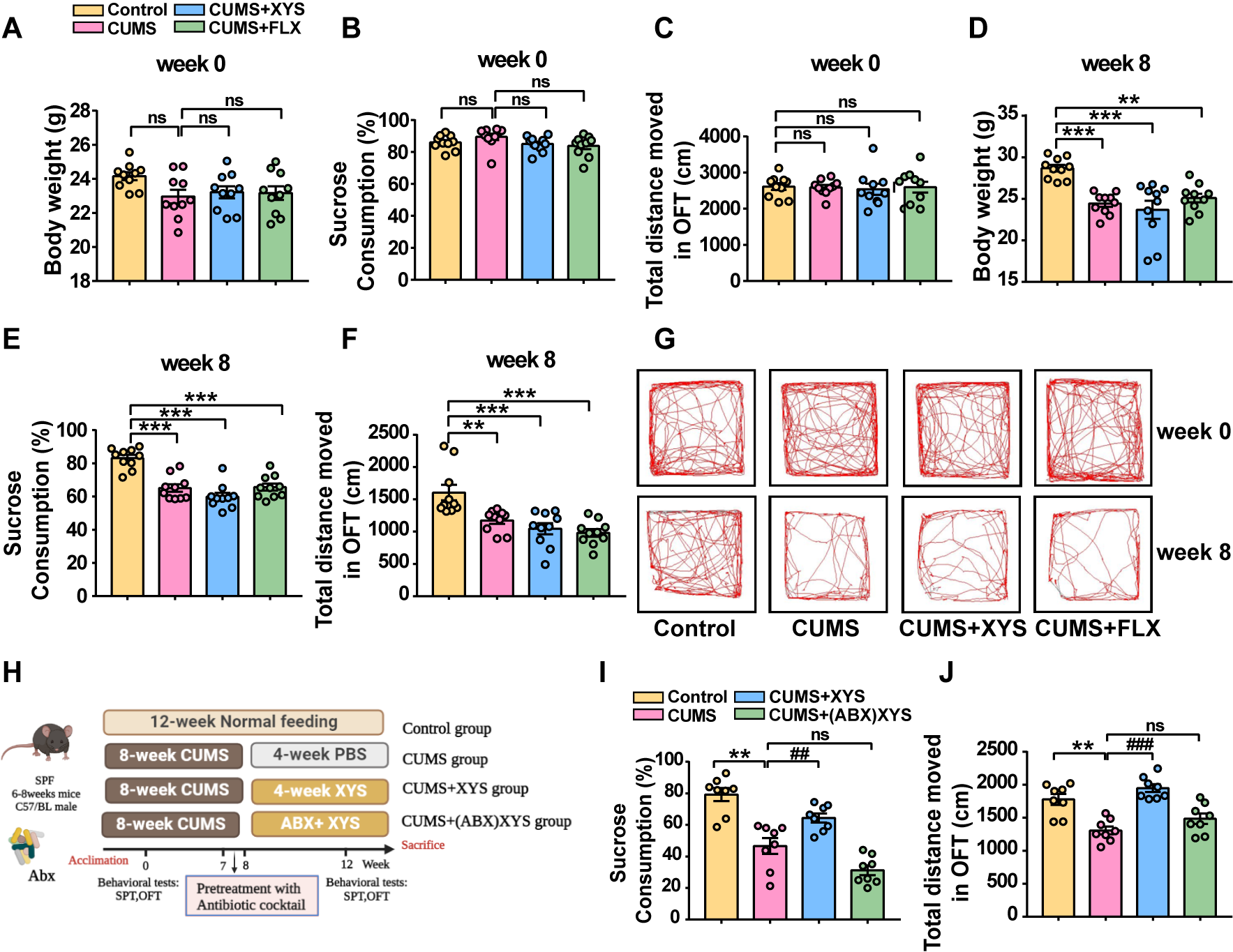

Supplement: Supplementary file 5 — Additional file 5: Supplement Fig. 1. Pretreatment with antibiotics attenuated the antidepressant effect of XYS. A: Body weight in week 0. B: SPT in week 0. C: OFT in week 0. D: Body weight in week 8. E: SPT in week 8. F: OFT in week 8. G: Movement trajectories of mice in OFT in week 0 and week 8. H: The study design of antibiotics interfering with the antidepressant effect of XYS. I: SPT. J: OFT. Data represent the mean ± SEM (n=10 per group). **P < 0.01, ***P < 0.001 versus the Control group; ##P< 0.01, ###P < 0.001 versus the CUMS group. [file 40168_2024_1756_MOESM4_ESM.tif]

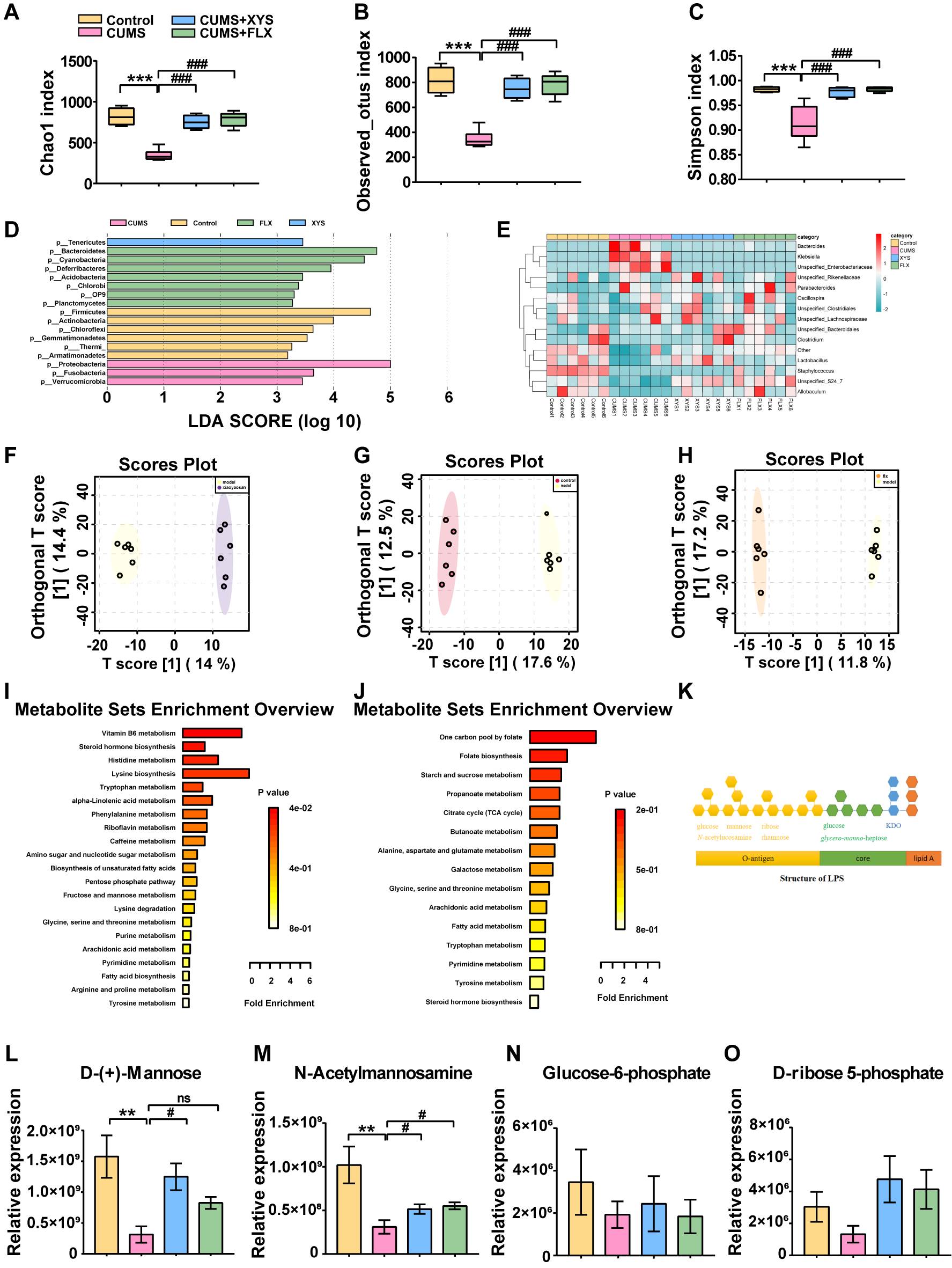

Supplement: Supplementary file 6 — Additional file 6: Supplement Fig. 2. Depression-like behaviors are accompanied by dysbiosis in gut homeostasis, and antidepressants restore gut homeostasis. Alpha diversity of A: chao1 index; B: observed index; C: simpson index. D: Analysis of differences in the microbial taxa at phylum level shown by LEfSe (linear discriminant analysis coupled with effect size measurements). E: Microbiota community of rats at the genus level, the relative abundance rates of each bacterial group. The relative abundance variation of each genus in normal group, CUMS group, XYS group and FLX group is indicated by a gradient of color from green (low) to red (high). Fecal bacteria distributed by Partial Least Squares Discriminant Analysis (PLS-DA) based on weighted UniFrac distance, between F: CUMS versus CUMS+XYS. G: Control versus CUMS. H: CUMS versus CUMS+FLX. KEGG enrichment results of differential metabolites in normal group, CUMS group, XYS group and FLX group of I: positive mode; J: negative mode. K: LPS-generated substrates. L: D-(+)-Mannose. M: N-Acetylmannosamine. N: Glucose-6-phosphate. O: D-ribose5-phosphate. Data represent the mean ± SEM (n=6 per group). ***P < 0.001 versus the Control group; ###P< 0.001 versus the CUMS group. [file 40168_2024_1756_MOESM5_ESM.tif]

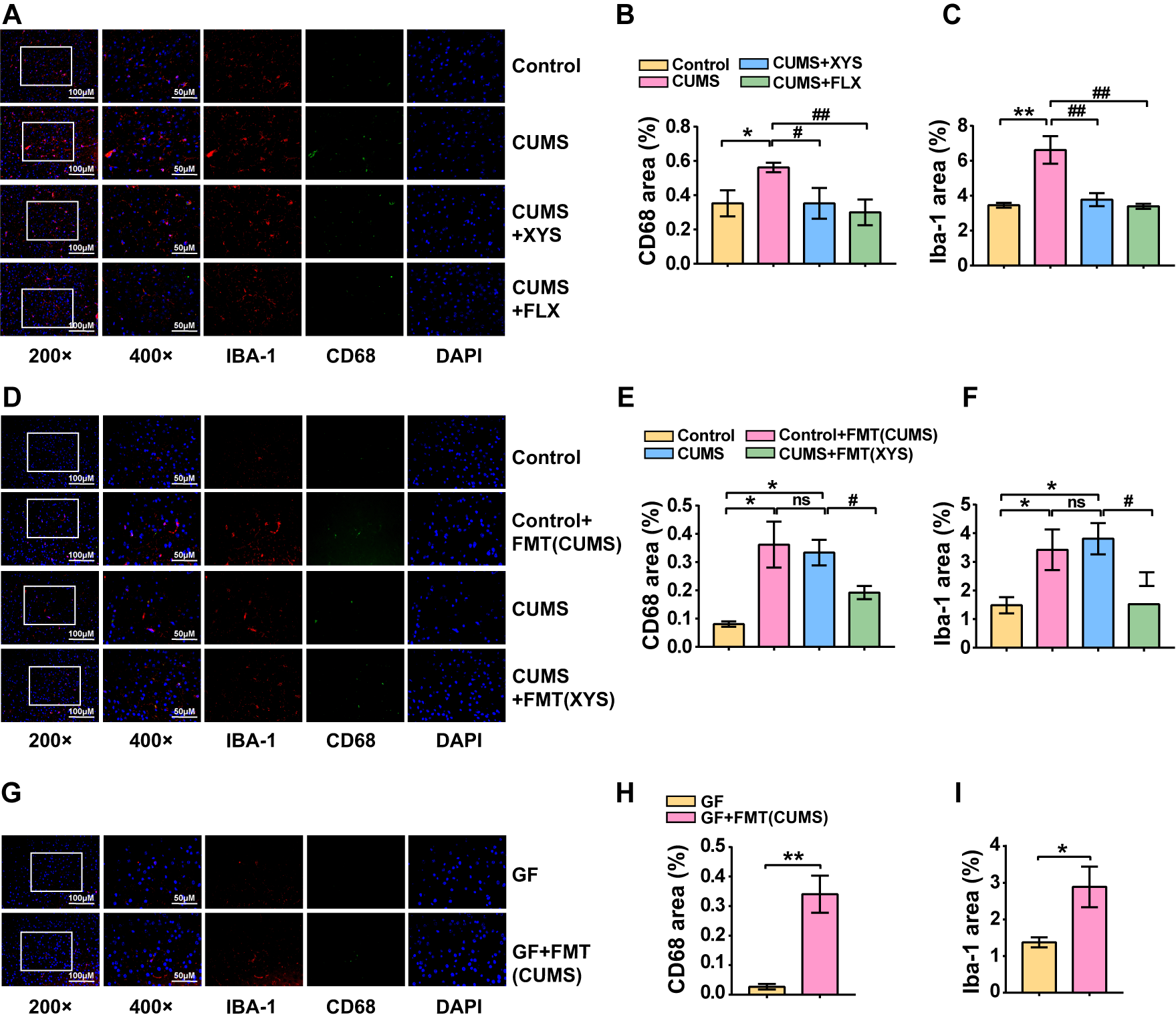

Supplement: Supplementary file 7 — Additional file 7: Supplement Fig. 3. Abnormal activation of microglia is important pathological manifestations of depression. A: Activation of CD68 and iba-1-labeled microglia (Control, CUMS, CUMS+XYS and CUMS+FLX). B: Activation of CD68 and iba-1-labeled microglia (Control, Control+FMT(CUMS), CUMS and CUMS+FMT(XYS)). C: Activation of CD68 and iba-1-labeled microglia (GF, GF+FMT(CUMS)). Data represent the mean ± SEM (n=3 per group). ***P< 0.001 versus the Control group; ###P < 0.001 versus the CUMS group. [file 40168_2024_1756_MOESM6_ESM.tif]

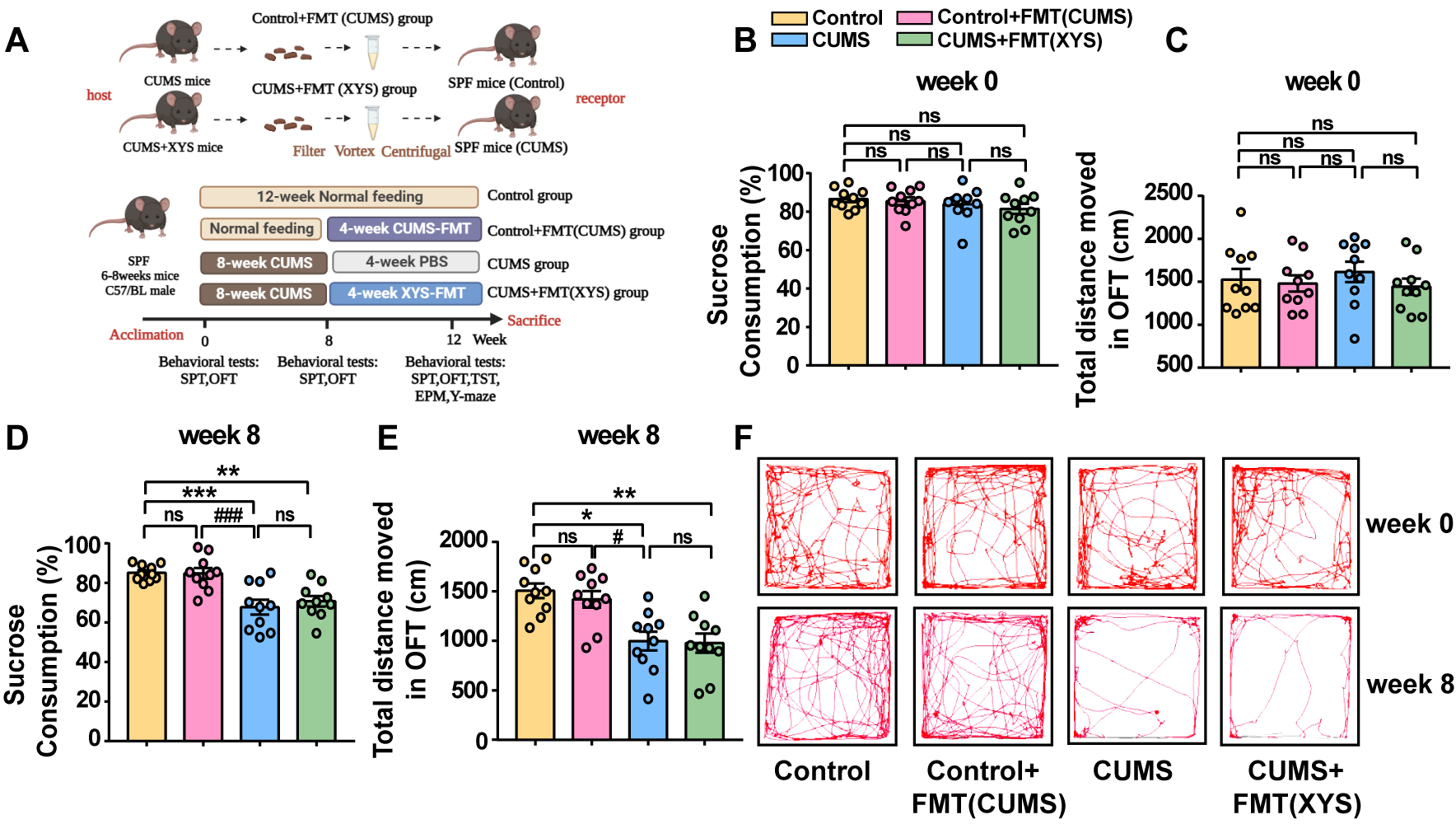

Supplement: Supplementary file 8 — Additional file 8: Supplement Fig. 4. Transplantation of dysregulated gut microbiota directly induces development of depression-like behaviors. A: Study design. B: SPT in week 0. C: SPT in week 8. D: OFT in week 0. E: OFT in week 8. F: Movement trajectories of mice in OFT. Data represent the mean ± SEM (n=10 per group). *P < 0.05, **P < 0.01, ***P< 0.001 versus the Control group; #P < 0.05, ##P< 0.01, ###P < 0.001 versus the CUMS group. [file 40168_2024_1756_MOESM7_ESM.tif]

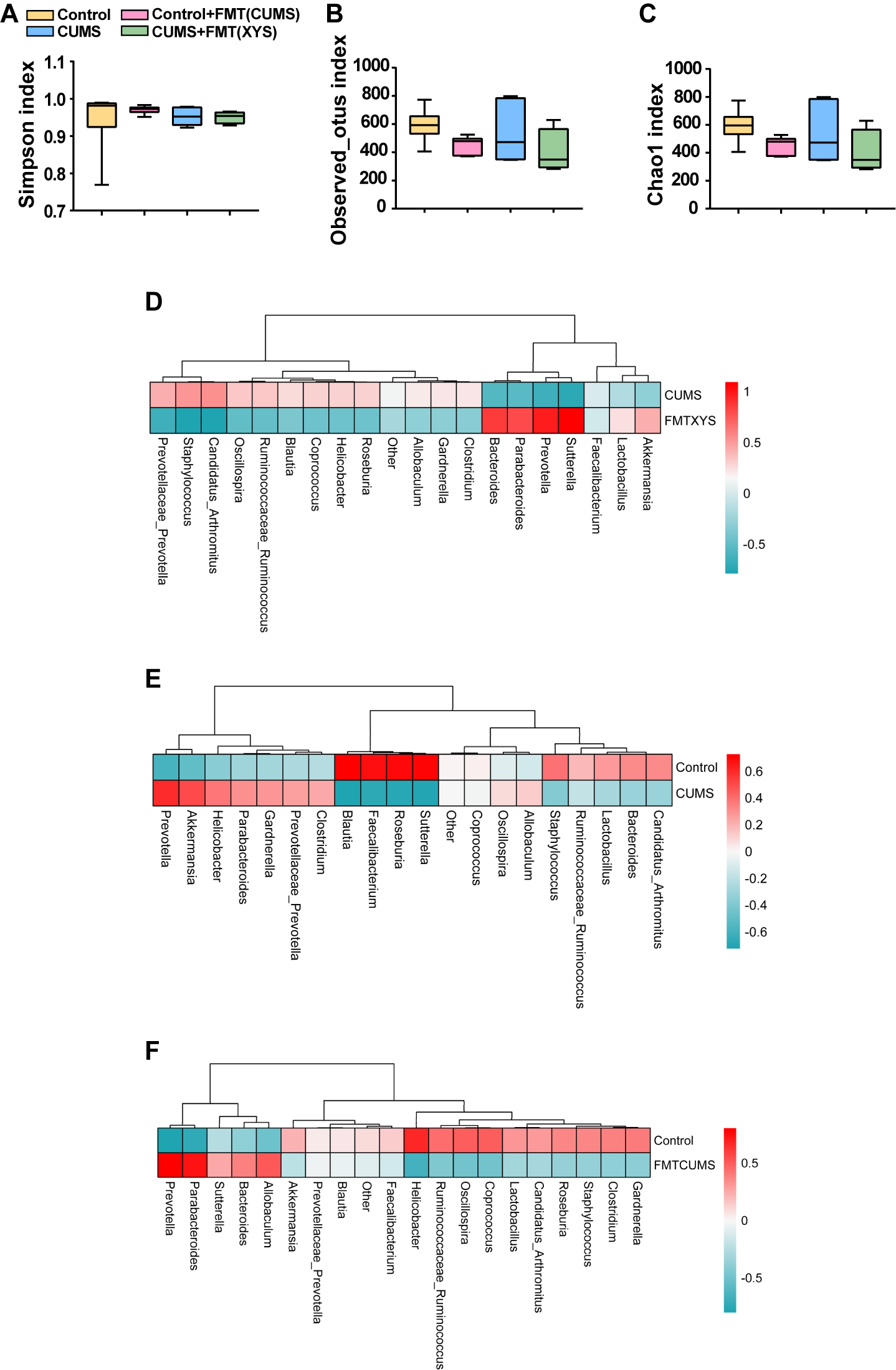

Supplement: Supplementary file 9 — Additional file 9: Supplement Fig. 5. CUMS-FMT mediated disturbance of the gut microbiota in mice; XYS-FMT regulated the composition of gut microbiota. Alpha diversity shown by A: simpson index. B: observed index. C: chao1 index. D: Gut microbiota change at genus level (Control versus CUMS). E: Gut microbiota change at genus level (Control versus Control+FMT(CUMS)). F: Gut microbiota change at genus level (CUMS versus CUMS+FMT(XYS)). [file 40168_2024_1756_MOESM8_ESM.tif]
